# Comparative genomic study of the *Penicillium* genus elucidates a diverse pangenome and 15 lateral gene transfer events

**DOI:** 10.1186/s43008-023-00108-7

**Published:** 2023-02-01

**Authors:** Celine Petersen, Trine Sørensen, Mikkel R. Nielsen, Teis E. Sondergaard, Jens L. Sørensen, David A. Fitzpatrick, Jens C. Frisvad, Kåre L. Nielsen

**Affiliations:** 1 Department of Chemistry and Bioscience, Aalborg University, Fredrik Bajers Vej 7H, 9220 Ålborg, Denmark; 2 Department of Chemistry and Bioscience, Aalborg University, Niels-Bohrs Vej 8, 6700 Esbjerg, Denmark; 3 Department of Biology, Maynooth University, Maynooth, W23 F2K8 Ireland; 4 Department of Biotechnology and Biomedicine, Technical University of Denmark, Søltofts Plads B221, 2800 Kgs, Lyngby, Denmark

**Keywords:** Long read sequencing, MinION, Filamentous fungi, *Penicillium*, Phylogenetics, Pangenome, Lateral gene transfer, Mycotoxins

## Abstract

The Penicillia are known to produce a wide range natural products—some with devastating outcome for the agricultural industry and others with unexploited potential in different applications. However, a large-scale overview of the biosynthetic potential of different species has been lacking. In this study, we sequenced 93 *Penicillium* isolates and, together with eleven published genomes that hold similar assembly characteristics, we established a species phylogeny as well as defining a *Penicillium* pangenome. A total of 5612 genes were shared between ≥ 98 isolates corresponding to approximately half of the average number of genes a *Penicillium* genome holds. We further identified 15 lateral gene transfer events that have occurred in this collection of *Penicillium* isolates, which might have played an important role, such as niche adaption, in the evolution of these fungi. The comprehensive characterization of the genomic diversity in the *Penicillium* genus supersedes single-reference genomes, which do not necessarily capture the entire genetic variation.

**Supplementary Information:**

The online version contains supplementary material available at 10.1186/s43008-023-00108-7.

## INTRODUCTION

The genus *Penicillium* is broad, ecologically diverse, and comprised of more than 483 known species. They are widespread and occupy various habitats including air, soil, indoor environments, vegetation, and food products (Houbraken et al. 2020; Visagie et al. 2014a). In nature, members of the genus are known decomposers of organic matter, which includes pre- and post-harvesting rotting of food crops (Frisvad and Samson 2004; Samson et al. 2019). On the other hand, the food industry utilizes the same decomposition, but also flavor and texture enhancing capability of some species in e.g., cheese production (Thom 1906; Nelson 1970; Karahadian et al. 1985; Giraud et al. 2010; Bodinaku et al. 2019) and fermented sausages (López-Díaz et al. 2001; Ludemann et al. 2010). A wide range of mycotoxins are produced by *Penicillium* (Frisvad et al. 2004; Perrone and Susca 2017*),* for example patulin, whose presence in fruits is tightly monitored and controlled (Perrone and Susca 2017). Naturally, the most famous extrolite produced by *Penicillium* spp. is penicillin, which revolutionized the medical treatment of bacterial diseases in the twentieth century (Fleming 1929). Generally, the *Penicillium* genus has received much attention and numerous studies screening for degradative abilities, exoenzyme production, and bioremediation but also for production of commercial antibiotics, cholesterol lowering agents, antioxidants, immunosuppressants etc. (Perrone and Susca 2017; Barrett et al. 2020; Schneider et al. 2016; Terrasan et al. 2010; Adsul et al. 2007; Li et al. 2007). A major challenge of such studies is that interesting compounds are often only produced under very specific conditions, which are very difficult to reproduce in laboratory settings. In contrast, whole genome sequencing opens the door to a more comprehensive view of the biosynthetic potential of the Penicillia. Advancements in long read sequencing technologies have provided opportunities to create affordable high-quality genome drafts (Petersen et al. 2022; Sørensen et al. 2014). Such high-quality genomes create higher comparative power when assessing the divergence of genes, pathways, and evolutionary development, which in turn allows more precise identification of species, orthologous genes, and gene clusters, and thus a better foundation for genome mining for novel compounds and enzymes, as well as a deeper understanding of evolutionary relationship. The use of a limited number of marker genes (e.g., ITS, *BenA*, *CaM,* and *RPB2*) sometimes leads to ambiguous results for closely related species due to little observed variation, and this can be overcome by the analysis of more comprehensive sets of genes, comprising hundreds or even thousands of conserved genes (Rokas et al. 2003). Incongruence may also arise in such datasets, but by increasing the number of genes and taxa, it can be minimized (Steenwyk et al. 2019). Furthermore, the structure of gene clusters, which is important for identifying orthologous gene clusters, synthesizing the same or similar compounds across species, can be analyzed in contiguous high-quality genome drafts (Blin et al. 2019).

In this study, we have sequenced and de novo assembled the genome drafts of 93 *Penicillium* isolates, of which 68 were considered individual species. The purpose is to reevaluate the established phylogeny with a more comprehensive gene set, as well as establish a *Penicillium* pangenome to gather insight into the entire gene pool and establish groups of orthologs. Furthermore, we analyzed the genome sequences for signs of lateral gene transfer (LGT) from bacteria to identify transfer events that might have an important role in the evolution of *Penicillium* genus.

## MATERIALS AND METHODS

### Penicillium isolates

91 *Penicillium* isolates were collected from the IBT Culture Collection of Fungi at the Technical University of Denmark (DTU, Denmark). *P. riverlandense* (CBS 135883) and *P. lagena* (CBS 129212) were purchased from CBS strain collection from Westerdijk Fungal Biodiversity Institute (Utrecht, The Netherlands). Further information regarding species and accession number of these isolates can be found in Additional file 1: Table S1. The isolates were grown and treated, sequenced, and assembled as described in Petersen et al*.* (2022).

Assemblies from an additional ten *Penicillium* isolates produced by others from long read sequencing data were downloaded from NCBI. These are: *P. camemberti* (FM013 = LCP06093 (NCBI ref. nr. PcamFM013r2_polished)), *P. capsulatum* (ATCC48735 (NCBI ref. nr. ASM94376v1)), *P. capsulatum* (LiaoWQ-2011 (NCBI ref. nr. ASM94377v1)), *P. digitatum* (DSM62840 (NCBI ref. nr. ASM1229554v2)), *P. digitatum* (PdW03 (NCBI ref. nr. ASM1676781v1)), *P. expansum* (R19 (NCBI ref. nr. Pexp_R19)), *P. oxalicum* (SGAir0226 (NCBI ref. nr. SGAir0226)), *P. polonicum* (F7 (NCBI ref. nr. ASM1346617v1)), *P. solitum* (RS1 (NCBI ref. nr. ASM95277v2)), and *P. solitum* (#12 (NCBI ref. nr. ASM1313803v1)). Furthermore, we included *P. aurantiogriseum* (IBT 35659 (NCBI ref. nr. ASM1997785v1)) that has recently been sequenced by our group (Petersen et al. 2022).

### High molecular weight DNA extraction and sequencing

High molecular weight DNA was extracted from the 93 *Penicillium* isolates using either Genomic Buffer Set (Qiagen, Germany) in combination with QIAGEN Genomic-Tips 20/G or using phenol–chloroform extraction in combination with QIAGEN Genomic-Tips 20/G as described in Petersen et al*.* (2022). Specific extraction methods are listed in Additional file 1: Table S1. Quality control of DNA was performed, followed by a removal of small DNA fragments to increase efficiency of sequencing, and finally another quality control was performed as described in Petersen et al*.* (2022). DNA Library preparations of two to four fungi were performed following the Native barcoding genomic DNA (EXP-NBD104, EXPNBD114, and SQK-LSK109) protocol from Oxford Nanopore Technologies (Oxford, UK) and the isolates were sequenced either on a R9.4.1 or R10.3 flow cell (Additional file 1: Table S1).

### Genome assembly and annotation

The raw reads were processed to generate 93 genome draft assemblies as described in Petersen et al*.* (Petersen et al. 2022). In short, Guppy (Technologies and pyguppyclient. Available from: 702https:, , github.com, nanoporetech, pyguppyclient(accessed 2022 September 23). 2022) was used to basecall, remove adaptors, and demultiplex the reads. Filtlong (Wick 2018) was used for trimming the reads for low confidence basecalls as well as short reads. Evaluation of the reads was performed with NanoPlot (Coster et al. 2018). Minimap2 (Li 2018) with default setting was used to map overlaps of the reads before the reads were assembled by Miniasm (Li 2016) using default setting. Subsequently, the assembly was polished using Racon (Vaser et al. 2017) and Medaka (Oxford Nanopore Technologies 2018) both with default setting. The different software versions used are listed in Additional file 1: Table S2 and trimming criteria can be found in Additional file 1: Table S1. Evaluation of assembly completeness was performed with Benchmarking Universal Single-Copy Orthologs (BUSCO) v5.0.0 using Eurotiales BUSCO dataset v10 (Manni et al. 2021).

FunGAP v1.1.0 (Min et al. 2017) was used to annotate the 93 genome draft assemblies and the 11 assemblies from NCBI. FunGAP was guided by an RNA-seq dataset that was generated by compiling RNA-seq data from several recent experiments of various *Penicillium* species: *P. bilaiae* (SRX2770370), *P. capsulatum* (SRX2022597), *P. digitatum* (SRX5587704), *P. glabrum* (SRX2770363), *P. novae-zelandiae* (SRX3064154), *P. solitum* (SRX5209310, SRX5209311), *P. steckii* (SRX3398614, SRX3398615, SRX3398616, SRX3398617, SRX3398618, SRX3398619), *P. swiecickii* (SRX3959645), *P. citreonigrum* (SRX7101841), and *P. subrubescens* (SRX7664426). One gigabase (gb) of sequences was randomly subsampled from each set and pooled. Protein sequences from *P. digitatum PHI26* (SAMN02399970), *P. oxalicum 114-2* (SAMN02981374), *P. freii* DAOM 242723 (SAMN03784687), and *P. flavigenum* (SAMN05200882) were downloaded from NCBI with the download_sister_orgs.py script from FunGAP to obtain the proteome database.

Secondary metabolite genes were predicted from the 104 proteomes using AntiSMASH v5.1 (Blin et al. 2019) and visualized using the R package ggplot (Wickham 2009). BiG-SCAPE (Navarro-Muñoz et al. 2020) was used to investigate gene cluster duplication of the biosynthetic gene cluster predicted by AntiSMASH in *P. soppii*. The Pfam database (Mistry et al. 2021) and InterProScan v5.38-76.0 (Jones et al. 2014) was used to functionally annotate the genes in each genome draft.

### Alignment and phylogeny.

BUSCO v5 analysis revealed 1,142 BUSCO families present in all 104 *Penicillium* isolates and two relevant outgroups (*Aspergillus fumigatus* (AF293) and *Aspergillus flavus* (NRRL3357)). Each BUSCO family was individually aligned with MUSCLE v3.8.1551 (Edgar 2004) and trimmed using trimAl v1.2 (Capella-Gutiérrez et al. 2009) with the parameter “-automated1” to remove poorly aligned regions. Trimmed alignments were concatenated together resulting in a supermatrix alignment of 885,360 amino acids. To speed up computation, phylogenetically uninformative sites were removed from the alignment, generating a final alignment of 364,162 amino acids. Maximum-likelihood phylogenetic reconstruction was performed using IQ-TREE v2.1.2 (Minh et al. 2020) with the JTT + F + R5 model, which was the best-fit model according to ModelFinder (Kalyaanamoorthy et al. 2017), and 100 bootstrap replicates were undertaken to infer branch support values.

Due to computational memory limitations, it was not possible to conduct whole genome alignment of all 104 genome sequences in a single analysis. Considering that additional information gained by whole genome alignment is most likely to be relevant for closely related species, four subtrees were pruned from the aforementioned phylogenetic tree (Additional file 2: Figure S1-S4). Whole genome alignment of the isolates in each subtree was then performed using CLC Genomics Workbench version 20.0 (Qiagen, Århus) using default setting. The resulting average nucleotide identity matrices—one for each subtree—was then used to generate four neighbour joining trees. The R package ggtree (Yu et al. 2017) was used to illustrate the figures.

### Mating type identification

The mating loci were identified from annotated genomes in GBK file format using the BLASTx algorithm with default settings in CLC Main Workbench version 7 (Qiagen, Århus). Coding sequences of the following genes were used as query: *Fusarium graminearum* PH-1 *MAT1-1-1* (FGSG_08892) and *MAT1-2-1* (FGSG_08893), *P. Roquefort* FM 317 *MAT1-1-1* (JX627318), *P. Roquefort* FM 164 *MAT1-2-1*, *SLA2* and *APN2* (KC469511), *P. chrysogenum* ATCC 28089 *MAT1-1-1* (AM904544), *P. chrysogenum NRRL* 1249B21 *MAT1-2-1* (AM904545). After identification of putative homologs, *P. alfredii* 34,128 *MAT1-1–1* (gene_00424), *APN2* (gene_00425), *SLA2* (gene_00423), and *P. antarticum* 31339 *MAT1-2-1* (gene_05584) were added to the list of query sequences and the BLASTx was repeated for every *Penicillium* genome. Synteny alignment plots were generated with the tBLASTx feature of EasyFig v2.1 (Sullivan et al. 2011) with BLAST options set to Min. length = 50 bp, Max. e-value = 0.001, and Min. Identity value = 50% for *P. malachiteum* or 25% for *P. macrosclerotiorum*.

### Pangenome construction

GET_HOMOLOGUES v3.3.2 with OMCL (Contreras-Moreira and Vinuesa 2013) was used to construct the *Penicillium* pangenome by clustering the 104 predicted proteomes into orthogroups with default setting. The pangenome was divided into core (genes present in all isolates), softcore (genes present in ≥ 95% of all isolates), shell (genes present in < 95% but > 2% of all isolates), and cloud (genes present in ≤ 2% of all isolates) genes. The pan and core gene size were estimated by random sampling of the genomes during the construction of the pangenome and fitted using Willenbrock model and Tettelin model for core and pan-genome, respectively. The estimate of the pan gene size was performed again with a subset of data (proteins with a length ≤ 150 aa, proteins with a length between 151 and 750 aa, proteins with a length between 751 and 1500 aa, and proteins with a length > 1500 aa) and all data to evaluate the difference in the saturation curve. However, DIAMOND (Buchfink et al. 2021) was used instead of BLASTp (Altschul et al. 1997) during similarity searches this time. A representative protein sequence of each orthogroup was chosen by taking the longest protein sequence within the average plus two standard deviations. Enrichment analysis of gene ontology (GO) was performed on these representatives, using GOATOOLS (Klopfenstein et al. 2018) and a false discovery rate of 0.05 was applied.

Estimation of the ratio of substitution rates at non-synonymous and synonymous sites (d_N_/d_S_) was calculated for each orthogroup in the softcore genome and each orthogroup with at least 10 genes in the shell genome. MUSCLE v5.0.1428 (Edgar 2021) was used to align the protein-coding sequences of each orthogroup. The alignments were converted to a nucleotide alignment that determined whether a substitution was caused by a synonymous or non-synonymous change using PAL2NAL (Suyama et al. 2006). CODEML from PAML (Yang 2007) was used to calculate pairwise d_N_/d_S_ values in each orthogroup with the site model M0. Lastly, median value for each orthogroup (9530 orthogroups for the shell genome and 5605 orthogroups for the softcore genome) was selected for further comparison. The values were log-normal transformed to find d_N_/d_S_ values that were significantly lower or higher than mean with a false discovery rate cutoff of 0.05. Mann Whitney U statistical testing was performed between core, softcore, shell, and cloud orthogroups for protein length, as well as softcore and shell orthogroups for d_N_/d_S_ values in R.

The pangenome was classified into euKaryotic Orthologous Groups (KOGs) by taking the representative protein sequence of each orthogroup used in the GO enrichment analysis and searching these against the KOG database (retrieved July 1st, 2022 from (http://ftp.ncbi.nih.gov/pub/mmdb/cdd/little_endian) using rpsblast (E value 0.01). Only the best hit from each orthogroup was used. If the KOG annotation had multiple KOG categories, all of them were included.

The pangenome matrix generated from GET_HOMOLOGUES was used to create a maximum likelihood tree using GET_PHYLOMAKERS (Vinuesa et al. 2018). Five independent IQ-TREE runs with ultrafast bootstrapping were performed. The best model was GTR2 + FO + R6. This tree is based on whether genes are present or absent. The tree was visualized and annotated using ITOL v5 (Letunic and Bork 2021).

Figures were illustrated with the R package ggplot (Wickham 2009) unless otherwise indicated.

### Identification of lateral gene transfer

The extent of LGT in each genome was assessed by combining all 104 proteomes together to give a query database of 1,244,528 proteins. Individual proteins were searched against a protein database representative of fully sequenced prokaryotic and eukaryotic species using BLASTp v2.10.1 + with an E value cut-off of 1e^−10^. This database consists of 1,698 genomes sampled from all three domains of life that had been used in a previous interdomain LGT analysis (McCarthy and Fitzpatrick 2016). *Penicillium* proteins that had a top hit to a bacterial species (3200 in total) were retained for a second round of database searches.

The 3200 *Penicillium* proteins were subsequently searched against the 20,671 Uniprot reference proteomes (Release 2022_01) using BLASTp with an E value cut-off set to 1e^−10^. Proteins with top hits to bacteria were again retained. A BLAST filter was applied where the query percentage identity to the top bacterial hits must be greater than 50% and the length of the query must be at least 80% of the subject (or vice versa). Furthermore, at least 80% of the top 200 hits for the query protein must come from bacteria. To maximise taxonomical coverage, a third round of database searches was then undertaken against NCBI’s non-redundant protein database (NR, last access 4th February 2022) to confirm a top hit to a bacterial source.

To avoid redundancy, candidate *Penicillium* LGT sequences were then grouped into 56 orthogroups using OrthoFinder (Emms and Kelly 2019). Representative and singleton sequences from these orthogroups were queried using BLASTp with an E value cutoff of 1e^−10^ against the Uniprot reference proteomes to locate homologs for phylogenetic analysis. For computational reasons, an arbitrary limit of 500 maximum hits per query sequence was imposed. We reconstructed 56 maximum likelihood (ML) phylogenetic trees for the candidate LGT families. Briefly, candidate LGT gene families were aligned using MUSCLE v3.8.1551. ML trees were inferred using IQ-TREE v2.1.2 with the TEST model and 100 bootstrap replicates. The resultant phylogenetic trees were inspected manually and visualized and annotated using ITOL v5. SignalP v6.0 (Teufel et al. 2022) was used to investigate whether the products of the LGT event genes were secreted.

## RESULTS

### Long read sequencing yielded high-quality genome drafts

**Fig. 1:**
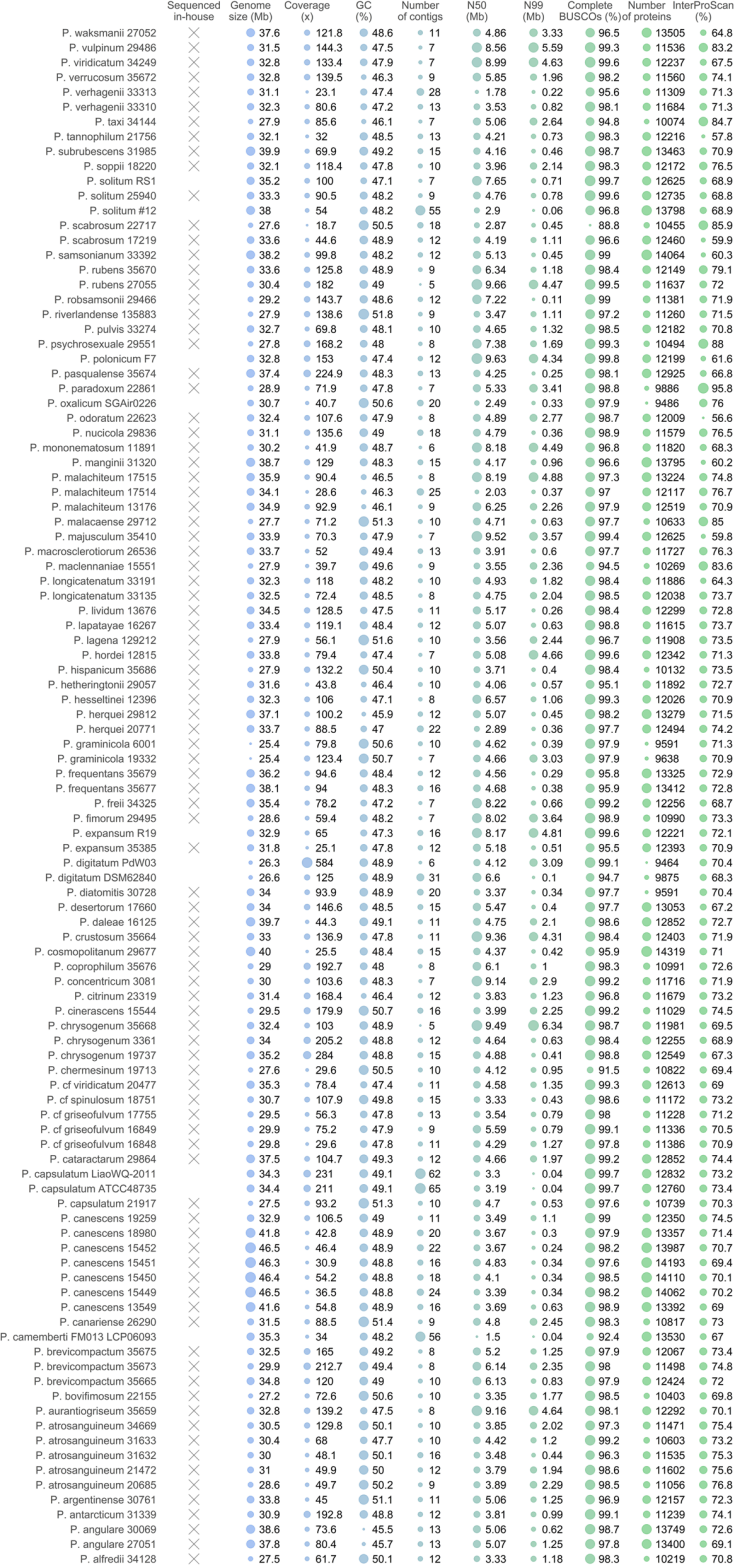
Genome characteristics. X indicates genomes sequenced in this study. Sequencing quality parameters and key genome numbers are illustrated by nine bubble plots. The bubble sizes are normalized and thus comparable within each column only, and not across columns

In this study, high molecular weight DNA from 93 *Penicillium* isolates was sequenced using the MinION long read sequencing platform, and de novo assembled into highly contiguous assemblies. The isolates were carefully chosen to ensure adequate phylogenetic/taxonomical coverage of the *Penicillium* genus. On average, 5.12 gb were sequenced per genome, of which, on average, 62.61% remained after filtering for minimal length and subsequently used in the assembly process (Additional file 1: Table S1). This corresponds to an average of 742,415 reads per genome. An average, 18.20% of these had the required minimal length to be included. The average assembled genome size was 33,27 Mb (25.4—46.5 Mb) and the number of contigs ranged from five to 28 (Fig. 1). To measure genome contiguity, we computed the N99. The N99 represents the minimum contig length needed to cover 99% of the genome. The average N99 across all genomes was 1.51 Mb. To evaluate genome completeness, BUSCO analysis was performed. An average of 97.87% complete BUSCOs were observed. Furthermore, 11 *Penicillium* genome assemblies made from long read data and thus with similar assembly characteristics were downloaded from NCBI, including *P. aurantiogriseum* that we recently sequenced (Petersen et al. 2022) (Fig. 1). All 104 *Penicillium* genome assemblies were annotated with FunGAP. The number of predicted proteins ranged from 9591 to 14,319 with an average of 11,976. The proportion of proteins assigned to protein superfamilies (containing a pfam domain) ranged from 56.6 to 95.8% per genome. Typically, secondary metabolite gene cluster contain one or a few core genes (e.g. polyketide synthases, non-ribosomal peptide synthases), as well a number of cluster specific genes (transporter, transcription factors etc.). The number of predicted secondary metabolite core genes range from 24 (in 24 biosynthesis gene clusters in *P. diatomitis*) to 112 (in 89 biosynthesis gene clusters in *P. soppii*) (Additional file 2: Figure S5). To ensure that we do not overestimate the number of unique biosynthetic gene clusters by including duplications, the gene clusters identified by antiSMASH in *P. soppii* were compared using BiG-SCAPE. We did not observe any clustering which would indicate duplications of gene clusters.

### Phylogenetics based on 1142 BUSCO genes reveals misidentification or contamination of accessions but generally supports previous suggested phylogentic trees

**Fig. 2:**
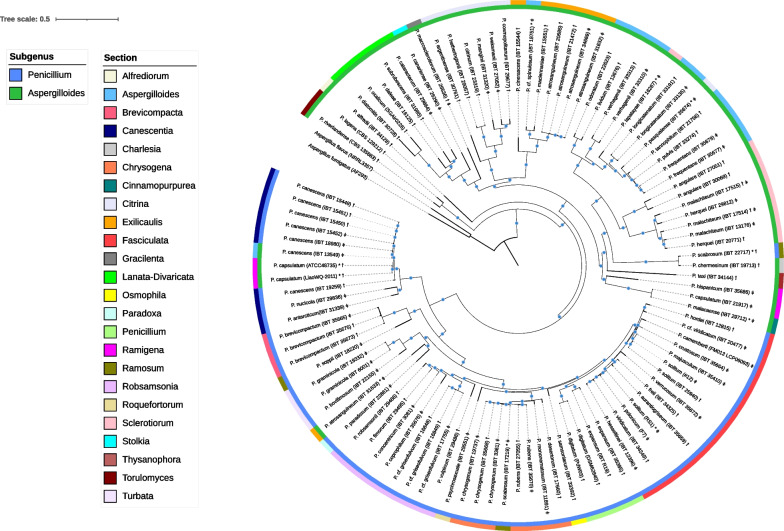
Representation of the phylogenetic relationship between the 104 *Penicillium* isolates. The phylogeny is based on 1142 BUSCO families. *Aspergillus fumigatus* AF293 and *Aspergillus flavus* NRRL 3357 were chosen as outgroups. The inner circle is colored according to the subgenera whereas the outer circle is colored according to sections as described in Houbraken et al. (2020). The blue circles state nodes with a bootstrap value of ≥ 95%. The tree scale is amino acid substitutions per site. *P. capsulatum* (ATCC48735)* and *P. solitum* (RS1)* have been reidentified as *P. canescens* (ATCC48735) and *P. polonicum* (RS1), respectively by Houbraken et al*.* (2021). Asterisk indicates isolates that have been misclassified. ꝉ and ǂ list mating type MAT1-1 and MAT1-2, respectively

To evaluate the phylogenetic relationship of the different *Penicillium* spp., we performed an alignment of 1,142 BUSCO gene shared by all 104 *Penicillium* isolates as well as two *Aspergillus* spp. outgroups. Annotations of subgenera and sections in accordance with Houbraken et al*.* (2020) were added (\* MERGEFORMAT Fig. 2). In general, the BUSCO genes yield a more robust tree with all nodes supported with bootstrap values of 100, expect two nodes with values 97 and 86. The lowest bootstrap value of 86 is found in the node separating the three highly similar *P. brevicompactum.* In comparison, bootstrap values in Houbraken et al. study vary from below 70 to 100, with many values below 85. A clear division of the two subgenera *Penicillium* and *Aspergilloides* was observed in the BUSCO tree. In this analysis, the phylogenetic placement of *P. riverlandense* and *P. lagena* (sect. *Torulomyces*) together with *P. alfredii* (sect. *Alfrediorum*) were distinct from the two subgenera. The placement of *P. alfredii* was consistent with that obtained by analysis of presence/absence of genes in the entire gene pool (Additional file 2: Figure S6). This, however, is not the case for *P. riverlandense* and *P. lagena*, which are placed in the middle of the subgenus *Aspergilloides*. Regardless, their placement outside of the two subgenera could indicate that *P. alfredii*, *P. riverlandense, and P. lagena* perhaps are closely related to ancestors of the rest of the *Penicillium* spp..

The BUSCO phylogeny assigned *P. capsulatum* (ATCC48735) and *P. capsulatum* (LiaoWQ-2011), together with *P. scabrosum* (IBT 22717) and *P. atrosanguineum* (IBT 31633) in unexpected subgenera. However, we believe these incongruences are likely to represent misidentifications or simple tissue collection inventory errors. Houbraken et al*.* (2021) reidentified *P. capsulatum* (ATCC48735) as a *P. canescens* (ATCC48735). This agrees with our observations (\* MERGEFORMAT Fig. 2, Additional file 2: Figure S3 and Additional file 2: Figure S6), and we further provide evidence that the same applies for *P. capsulatum* (LiaoWQ-2011). Thus, *P. capsulatum* (LiaoWQ-2011) should be named *P. canescens* (LiaoWQ-2011). Additionally, *P. solitum* (RS1) have been reidentified as *P. polonicum* (RS1) (Houbraken et al. 2021), and in agreement with this, our conserved BUSCO phylogeny shows close relationship to *P. polonicum*. *P. scabrosum* (IBT 22717) clustered close to *P. chermesinum* and is therefore most likely not a *P. scabrosum*, but instead one species of the sect. *Charlesia*. In addition, the other *P. scabrosum* analyzed (IBT 17219) is in sister-group relationship with *P. chrysogenum*. *P. atrosanguineum* (IBT 31633) does not cluster with the other *P. atrosanguineum* strains but close to *P. paradoxum* and should likely belong to sect. *Paradoxa*. In all cases, consistent phylogeny was observed based on presence/absence of genes (Additional file 2: Figure S6) as well as based on whole genome alignment (Additional file 2: Figure S1-S4).

The BUSCO phylogeny and the placement of subgenera displays a high degree of congruence with a previous phylogenetic analysis of the *Penicillium* genus (Houbraken et al. 2020); that analysis was based on a set of three gene markers (*BenA*, *CaM* and *RPB2*). The *Ramosum* sect. consisting of *P. soppii*, *P. scabrosum* (IBT 17219), and *P. scabrosum* (IBT 22717) were distinct from each other in the conserved BUSCO gene phylogeny. As mentioned before, we believe a misidentification of the two *P. scabrosum* accessions (IBT 22717, IBT17219) has occurred (\* MERGEFORMAT Fig. 2, Additional file 2: Figure S1-4, and Additional file 2: Figure S6). Conversely, the phylogenetic position of *P. soppii* was in congruence with Houbraken et al*. *(2020), and it is presumed to show the correct phylogenetic position for the sect. *Ramosum*. According to Houbraken et al*.* (2020), *P. samsoniarum* (sect. *Osmophila*) and *P. psychrosexuale* (sect. *Roquefortorum*) should cluster together, but this is not the case in the phylogenetic based on the conserved BUSCO gene set (\* MERGEFORMAT Fig. 2). However, the phylogeny based on whole genome alignment showed that *P. samsoniarum* is closely related to *P. psychrosexuale*. Given the expected increased power of whole genome alignment to elucidate close relationships, we believe that *P. samsoniarum* and *P. psychrosexuale* are more closely related phylogenetically than suggested in conserved BUSCO phylogeny. Furthermore, the clustering pattern of sect. *Charlesia*, *Ramigena*, and *Thysanophora* was slightly different compared to Houbraken et al*.* (2020). We further observed that the *Cinnamopurpurea* sect. consisting of only *P. malacaense* was included in the *Ramigena* sect. due to the close clustering with *P. capsulatum* (IBT 21917). We propose that *P. malacaense* is a *P. capsulatum* spp. resulting in the *Cinnamopurpurea* sect. not being represented in the phylogeny at all. A few other examples of inconsistencies can likewise be observed in the sect. *Exilicaulis* and *Aspergilloides*: *P. pasqualense*, *P. lapatayae*, and *P. cf. Spinulosum* (\* MERGEFORMAT Fig. 2).

Given the aforementioned observations *P. atrosanguineum* (IBT 31633), *P. scabrosum* (IBT 17219), *P. scabrosum* (IBT 22717), *P. malacaense* (IBT 29712), *P. pasqualense* (IBT 35674), *P. lapatayae* (IBT 16267), and *P. cf. Spinulosum* (IBT 18751) should for now be reidentified as *Penicillium* sp. (IBT 31633x), *P. chrysogenum* (IBT 17219x), *P. chermesinum* (IBT 22717x), *P. capsulatum* (IBT 29712x), *Penicillium* sp. (IBT 35674x), *Penicillium* sp. (IBT 16267x), and *Penicillium* sp. (IBT 18751x), respectively.

Penicillia can be heterothallic or homothallic. This is determined by the two loci MAT1-1 and MAT1-2. Heterothallic strains harbor either MAT1-1 or MAT1-2 locus whereas homothallic strains harbor both loci next to each other (Dyer and Kück 2017; Dyer et al. 2016). MAT1-1 and MAT1-2 can be found across the phylogeny (\* MERGEFORMAT Fig. 2). The majority of the *Penicillium* isolates share the same gene structure with the mating loci located between the *SLA2* gene and the *APN2* gene. A total of 99 isolates are heterothallic as expected, with 53 isolates carrying the *MAT1-1-1* gene and 46 isolates carrying the *MAT1-2-1* gene. Surprisingly, however, both *MAT1-1-1* and *MAT1-2-1* gene can be found in *P. macrosclerotiorum* (IBT 26536), *P. malachiteum* (IBT 17514), *and P. malachiteum* (IBT 17515) making them homothallic (Figure S7-S8). By manual inspection of read data we were able to identify single reads spanning both loci, confirming that both MAT1-1-1 and MAT1-2-1 genes are present in the same genome molecule.

### The Penicillium pangenome contains a large accessory gene pool

**Fig. 3:**
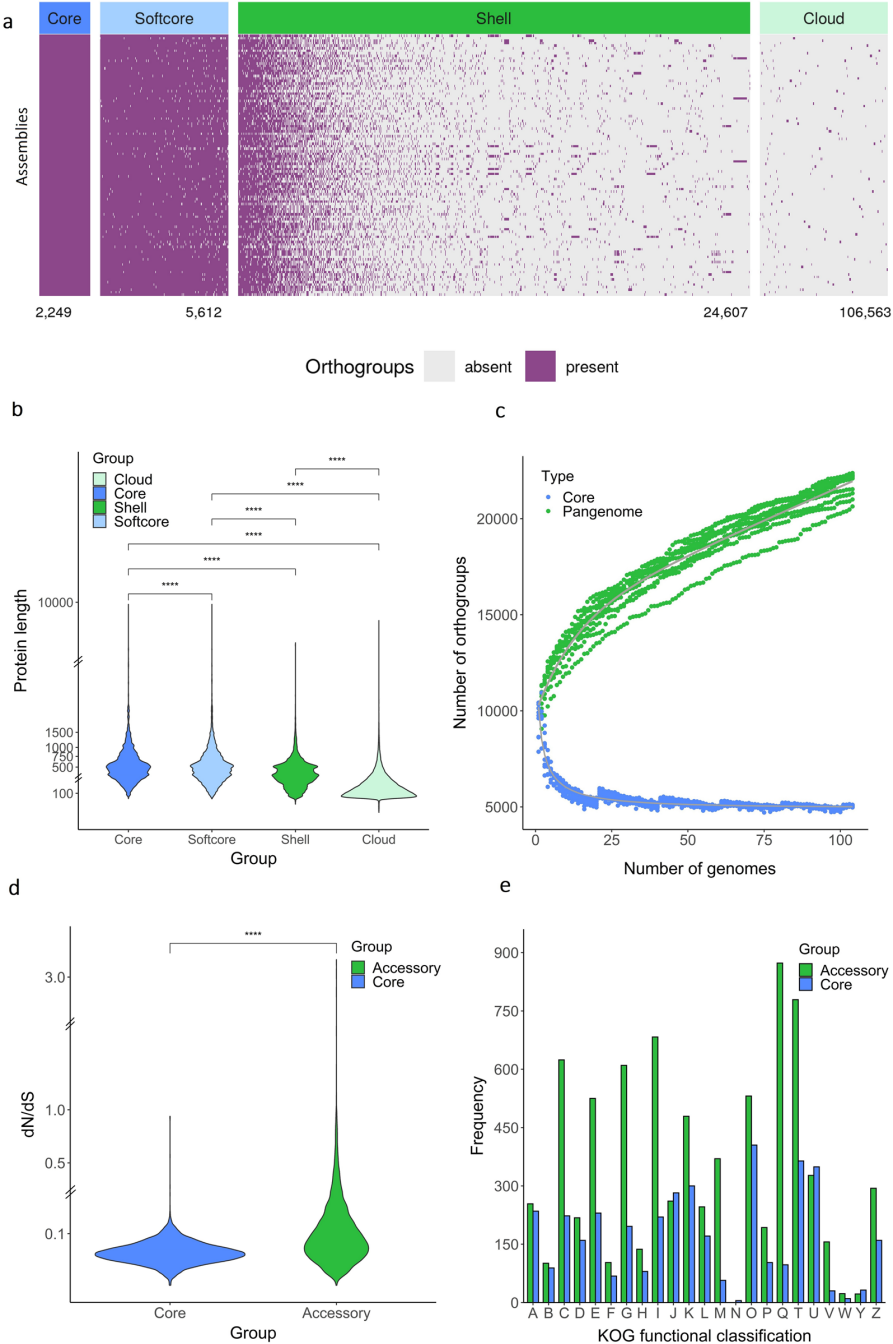
Pangenome characteristics. **a** The distribution of orthogroups in the core, softcore, shell, and cloud genome of *Penicillium*. The x-axis lists the different orthogroups whereas the y-axis lists the different assemblies. Purple and grey list whether the orthogroup is present or absent, respectively. The number under each panel lists the number of orthogroups in the different part of the pangenome. The panels were scaled after the number of orthogroups. Thus, softcore genome is approximately two and half times larger than core genome, whereas shell genome is approximately four times larger than softcore genome. The exception is the cloud genome that should be four times larger than shell genome, but instead is four times smaller. **b** The distribution of lengths of all protein sequences in the pangenome. The y-axis is discontinuous. All distributions were significantly different from each other (two-sided Mann–Whitney U-test) as indicated by stars. **c** The cumulative size of the *Penicillium* gene pool. The analysis was performed ten times with random addition of genomes. **d** The distribution of the median d_N_/d_S_ value for each orthogroup. The y-axis is discontinuous. The two distributions are significantly different from each other as indicated by stars corresponding to a p value of < 2 × 10^−16^ using two-sided Mann–Whitney U-test. **e** KOG functional classification of the pangenome. Only the best hit from each orthogroup was included and if this hit had multiple categories, all of them are plotted. The R and S category were discarded in the plot. A, RNA processing and modification; B, chromatin structure and dynamics; C, energy production and conversion; D, cell cycle control, cell division and chromosome partitioning; E, amino-acid transport and metabolism; F, nucleotide transport and metabolism; G, carbohydrate transport and metabolism; H, coenzyme transport and metabolism; I, lipid transport and metabolism; J, translation, ribosomal structure, and biogenesis; K, transcription; L, replication, recombination, and repair; M, cell wall/membrane/envelope biogenesis; N, cell motility; O, post-translational modification, protein turnover, and chaperones; P, inorganic ion transport and metabolism; Q, secondary metabolites biosynthesis, transport, and catabolism; T, signal transduction mechanisms; U, intracellular trafficking, secretion, and vesicular transport; V, defense mechanisms; W, extracellular structures; Y, nuclear structure; and Z, cytoskeleton

The predicted proteomes from the 104 genome assemblies were used to define and characterize the pangenome of *Penicillium* to uncover the genetic diversity of the genus. A pangenome is defined as the entire set of genes of a strain, species, or genus. It can be divided into core and accessory genome, which are orthogroups present in all isolates and orthogroups that are not shared by all isolates, respectively. As part of the core genome, 2249 orthogroups were defined since these were present in all isolates, whereas 5612 orthogroups were identified as part of the softcore genome, a subdivision of core genome where an orthogroup can be found in at least 98 isolates (95% of 104 isolates) (Fig. 3a). The softcore genome also includes the orthogroups from core genome. The accessory genome can be further divided into a shell genome corresponding to orthogroups found in 3–97 isolates and a cloud genome corresponding to orthogroups found in two isolates or singleton genes. Respectively, 24,607 and 106,563 orthogroups belongs to shell and cloud genome. Due to their wide distribution within the genus, orthogroups belonging to the core/softcore and shell genomes likely represent true protein coding genes, conversely, a proportion of the cloud genome is likely to not represent functional genes, but instead gene fragments, pseudogenes, and errors during gene calling. This is supported by the observation that the median lengths of every protein sequence in the core (486 aa), softcore (471 aa), and shell (356 aa) are rather similar, whereas the median length of the cloud (145 aa) orthogroups is much lower. (Fig. 3b). For this reason, the shell genome likely best describes the accessory genome and further work with the accessory genome was performed using the shell genome only. Henceforth, the term accessory genome will be used instead of shell. Furthermore, from the observed BUSCO completeness of 97.87% we can expect that the chance of observing a true core genome orthogroup in all genomes is only 0.9787^104^ = 10.66%, rendering the strict definition of core genome meaningless. As a consequence, the softcore genome is taken as representing the true core genome and henceforth the term core genome will be used.

Predicted proteins with a length > 150 aa were used to estimate the size of the pan- and core genome size by random addition of genome sequences. This process was replicated 10 times. The saturation of the core genome indicates a relatively closed pangenome, meaning that the addition of new genome assemblies did not substantially decrease the number of orthogroups (Fig. 3c) in the core genome. In contrast, addition of new genome sequences to the pangenome adds new orthogroups to the gene pool, indicating an open pangenome. The same analysis was also performed on subsets of data that were extracted based on protein length from Fig. 3b. From here, it is clear that protein sequences ≤ 150 aa did not show any saturation whereas three subsets, 151–750 aa, 751–1500 aa, and > 1500 aa showed saturation as protein length increased (Additional file 2: Figure S9). It is likely that predicted proteins with a length < 150 aa contains a larger proportion of gene fragments, pseudogenes, and other errors than larger proteins. Taken together, these results show that while the core genome of *Penicillium* is well characterized in this pangenome model, the accessory genome is less comprehensively described and while showing signs of saturation, considerable gene, and therefore potential for metabolic diversity, is not included in the present pangenome model. Regardless, the large accessory genome observed agrees with the observed diversity of *Penicillium* habitats, enabling isolates to survive in diverse environmental niches.

The distribution of gene ontology (GO) terms between the core genes and the entire gene pool as well as the accessory genes and the entire gene pool was also analyzed (Additional file 3). As expected, GO terms involved in general housekeeping such as development and basic cellular function, were significantly enriched in the core genome, and included “cellular metabolic process”, “protein binding”, and “ATP binding”; in total, 140 GO terms were enriched. Conversely, “transmembrane transport”, “monooxygenase activity”, “heme binding”, and “FAD binding” etc. were significantly unrepresented in the core genome; in total, 12 GO terms were underrepresented. The opposite was observed in the accessory genome where GO terms which originate from general housekeeping functions were significantly underrepresented; in total, 603 GO terms were underrepresented. Furthermore, the accessory genome was significantly enriched for “secondary metabolite biosynthetic process” together with “mycotoxin biosynthetic process” and “heme binding”; in total, 25 GO were enriched. This validates the assumption that the core genome controls general housekeeping systems that are needed in all members of the genus, whereas the accessory genome varies within the genus and enables differential response to external stimuli, including the capacity to synthesize commercially interesting secondary metabolites and enzymes. In agreement with this analysis, heme binding was also observed to be significantly enriched in the accessory genome of *Aspergillus fumigatus* (Barber et al. 2021). Heme is found in decaying organic matter and can either be utilized as cofactors in many enzymes directly, or converted to iron (Kornitzer and Roy 2020). Additionally, “transition metal ion binding” and “zinc ion binding” were likewise significantly enriched in the accessory genome together with “monooxygenase activity” and “oxidoreductase activity”, which are tailoring enzymes and their cofactors involved in the biosynthesis of several secondary metabolites. However, significant underrepresentation of these tailoring enzymes and their cofactors was observed in the core genome, indicating the versatility of secondary metabolites across the genus of *Penicillium*.

The rate of non-synonymous-to-synonymous substitutions (d_N_/d_S_) for each orthogroup were calculated to reveal evolutionary selection in the core and accessory genome (Fig. 3d). Overall, both core and accessory genes may be under a general trend of purifying selection as indicated by d_N_/d_S_ values lower than one. This observation is in line with another analysis on a pangenome from a filamentous fungus (Wyka et al. 2022). Not surprisingly, lower average d_n_/d_s_ ratio was observed in core genes compared to accessory genes. The core genome consists of conserved genes that are generally under high purifying selection whereas the purifying selective pressure is expected to be less on the accessory genome. Since only a few genes were found to be under positive selection (d_N_/d_S_ values > 1), d_N_/d_S_ values significantly less or higher than mean in both the core and accessory genome were found (Additional file 4—Table S11-14) and GO enrichment analyzes were performed on these genes (Additional file 4: Table S15). Within the core genome, 124 orthogroups were found to have d_N_/d_S_ values significantly less than the mean, and seven orthogroups were found to have significantly higher values than mean. Within the accessory genomes, 45 and 27 orthogroups were found to have significantly less or higher d_N_/d_S_ values than the mean values, respectively. Considering the core genome, no GO enrichment was observed among the seven orthogroups that had d_N_/d_S_ values significantly higher than the mean, simply because of the low number of orthogroups. However, one notable orthogroup encodes an ABC transporter. Fungal ABC transporter are involved in the efflux of, amongst others, natural toxic compounds such as xenobiotics (Víglaš and Olejníková 2021). Positive selection of such genes has been observed within competing organisms in the same ecological niches or in symbiotic relationships (Derbyshire 2020; Vallender and Lahn 2004). Twenty-five GO terms were enriched between the 124 orthogroups that had d_N_/d_S_ values significantly less than the mean, with the majority related to “GTP binding” and “GTPases” such as genes encoding RAS family, ADP-ribosylation factor family, and septins. Furthermore, enrichment involving “transferase activity, transferring phosphorus-containing groups” was observed within genes encoding protein kinase domain, 4′-phosphopantetheinyl transferase superfamily, FGGY family of carbohydrate kinases, and RNA polymerase. None of the 27 orthogroups that had significantly larger d_N_/d_S_ values than the mean in the accessory genome had any GO functional annotation. Further, NCBI database searches of these 27 orthogroups returned five genes with results (zinc finger, protein kinase-like domain, and rRNA intron-encoded homing endonuclease), four genes with no significant results, and the rest were results that matched hypothetical proteins. None of these hits were immediately noteworthy, nor consistent, and we therefore conclude that no functional categories under positive selection can be identified. Further, we find it likely that the majority of these genes are in fact not truly functional genes, but pseudogenes and gene fragments that do not give rise to functional gene products. Such nonfunctional genes are not expected to be under the same selective pressure as functional genes. In contrast, enrichment towards “mitotic spindle” (two orthogroups) was observed in the 45 orthogroups that had significantly lower d_N_/d_S_ values than mean in the accessory genome. The orthogroups encode DASH complex subunit Dad4. However, it may not be surprising that a component of the DASH complex, a central part of the mitotic spindle, apparently diverse enough to fall in the accessory genome category in our pangenome analysis, is among the genes under most purifying selection in this category, considering the importance of the mitotic spindle to one of the most essential biological processes—mitosis.

A KOG functional classification of the pangenome showed that 77.5% and 37.3% of the orthogroups belonging to core and accessory genome, respectively, could be classified (Fig. 3e). The core genome contained 25% metabolic genes and 54.4% non-metabolic genes whereas the accessory genome was more equally distributed with 36.2% metabolic genes and 39.2% non-metabolic genes. “Posttranslational modification, protein turnover, chaperones” (O) was mostly represented in the core genome along with “signal transduction mechanisms” (T) and “intracellular trafficking, secretion, and vesicular transport” (U). As expected, the accessory genome was dominated by “secondary metabolites biosynthesis, transport and catabolism” (Q) followed by “signal transduction mechanisms” (T) and “lipid transport and metabolism” (I).

### Fiftteen unique LGT events have happen in Penicillia

In order to locate putative LGT events, we concatenated all 104 *Penicillium* proteomes, yielding a query database of 1,244,528 proteins. Each query protein was searched against a taxonomically diverse sequence database and queries with top hits to bacterial species were retained for further searches against the UniProt reference proteomes and NCBI’s non-redundant protein database. In total, 625 *Penicillium* proteins were found to have a top hit to a bacterial species. These 625 proteins were grouped into 56 orthogroups, and phylogenetic trees were reconstructed for each orthogroup and their homologs (see Methods). Individual phylogenetic trees were manually inspected to confirm incongruent relationships relative to the species phylogeny*.* A study by Wang and Ruan et al. ( 2020) have identified ancestral LGT events into other fungal genera. Therefore, we discarded phylogenies that displayed evidence of ancestral LGT into other fungal genera and found support for 15 unique LGT events in the *Penicillium* genus only (Additional file 2: Figure S10-24, \* MERGEFORMAT Table 1, Additional file 5). These 15 events represent 139 genes in total (\* MERGEFORMAT Table 1 and Additional file 5) and are distributed across 82 of the 104 *Penicillium* genomes under consideration (Figure S25). Furthermore, when multiple strains per species are excluded, these 15 events are located in 57 unique *Penicillium* species. Overall, 95 of the 139 LGT genes lack introns (68%) compared to 287,428 of all 1,244,528 (23.1%) *Penicillium* proteins in our dataset, indicating the LGT genes have a different intron profile relative to the genomes they are located in. Based on SignalP v6.0 predictions, none ofthe products of the LGT event genes were secreted.

**Table 1:** Functional annotation of LGT events

	# LGT proteins in Tree	# Genomes represented	Annotation	Average length of LGT proteins	Average % ID to bacterial species	Average # introns
LGT1^1^	14	14	Glycoside hydrolase 105 family protein	389	59	1.1
LGT2	21	21	FAD-binding protein	524	62	0.1
LGT3	6	6	10 kDa chaperonin	96	73	3.0
LGT4	18	10	Amidohydro_3 domain-containing protein	562	54	0.4
LGT5	2	2	Zinc-binding alcohol dehydrogenase	331	61	0.5
LGT6	2	2	Elongation factor Tu	481	69	0.0
LGT7^2^	52	51	Short-chain dehydrogenase	227	79	0.0
LGT8^3^	13	12	SMI1_KNR4 domain-containing protein	187	43	0.8
LGT9	4	4	Acyl carrier protein	78	78	3.0
LGT10	2	2	Peptidyl-prolyl cis–trans isomerase	162	61	0.0
LGT11	1	1	NAD-dependent epimerase/dehydratase	304	61	0.0
LGT12	1	1	NADP octopine/nopaline dehydrogenase	362	51	0.0
LGT13	1	1	DUF4976 domain-containing protein	483	62	1.0
LGT14	1	1	Cold-shock protein	71	66	0.0
LGT15	1	1	Extracellular solute-binding protein	278	60	0.0

^1,2,3^ correspond to HGT56, HGT1, and HGT18 previously located by Wang and Ruan et al. (Wang and Ruan 2020)

Ten of the putative LGT genes (LGT1-10, \* MERGEFORMAT Table 1) are located in > 2 genomes and are considered high confidence inferences. Three of these LGT events (LGT1,7 & 8) have been reported previously (Wang and Ruan 2020). The number of LGT events per genome ranges between 0 and 3 (Additional file 2: Figure S25). LGT1 and LGT8 are the most ancestral transfers and occurred before the speciation of the majority of *Penicillium* species (Additional file 2: Figure S25); however, their patchy phylogenetic distribution is indicative of extensive gene loss in individual species and lineages. Alternatively, other LGT events, such as LGT2 and LGT7, occurred along specific lineages and have been maintained. The majority of LGT genes are single copy, but LGT4 shows evidence of a tandem duplication event, and eight of the 10 genomes contain a paralog. Synteny (not shown) and phylogenetic analysis (Additional file 2: Figure S13) indicate that this duplication occurred in the ancestor of the species that contain these genes (Additional file 2: Figure S25).

## DISCUSSION

Vast improvements in sequencing technologies have occurred in recent time, including long read DNA sequencing technologies such as Oxford Nanopore Technologies. In turn, these developments facilitate increased insight into fungal genetics by permitting mid or high throughput sequencing projects of groups of organisms. In this study, we have sequenced, de novo assembled, and annotated 93 *Penicillium* isolates and provided a significant contribution of high contiguity and completeness to the number of publicly available *Penicillium* genome drafts (358 genome drafts were available at NCBI in October 2022 of which 15 originate from long read sequencing data). While the genomes are of high contiguity, completeness, and elucidate the potential *Penicillium* spp. have to produce a vast number of secondary metabolites with pharmaceutical, agricultural, or industrial value, a note of caution is warranted. Sequencing errors are inherent to DNA sequencing and the error profile of Oxford Nanopore Technologies is not completely random but biased towards indel errors in homopolymer regions. This problem cannot be easily overcome by increasing read coverage but requires different sequencing technology, such as Illumina short-reads (Stoler and Nekrutenko 2021) or newer versions of Oxford Nanopore Technologies (Sereika et al. 2022).

A benefit of the use of whole genome data is that it facilitates the use of genome-scale phylogeny instead of a few marker genes only. The inclusion of more data could potentially add more resolution and power to the analysis. However, it would be expected that, long range phylogenetic relationships are better represented by hyperconserved regions of the genome, e.g. a limited set of hyperconserved marker genes, since more variable parts would undergo multiple substitutions acting as “noise” in long range analysis. Conversely, the variable parts of the genome are better at resolving evolutionary relationships of closely related organisms, and it is to the analysis of these variable parts that whole genome alignment can contribute. Further, a relevant set of conserved genes, which can serve as “intermediate” variable genes, is the BUSCO gene set. In this paper, we have conducted phylogenetic analysis with the whole genome, BUSCO gene set, as well as presence/absence of orthologous genes (see Methods for details) and compared it with already published phylogeny based on three marker genes (Houbraken et al. 2020). In general, the four different approaches are highly congruent with one other, indicating convergence of the phylogenetic analyses to the true evolutionary relationship of the *Penicillium* genomes. There are, however, a few noteworthy exceptions. The unique placement of *P. alfredii*, *P. lagena*, and *P. riverlandense* outside the two subgenera is unexcepted and stands in contrary to what was observed by Houbraken et al*.* (2020). *P. alfredii* was isolated from house dust on an island in the Federated States of Micronesia (Visagie et al. 2014b). *P. lagena and P. riverlandense* were isolated from soil in the USA, and bract from *Protea repens* infructescens in South Africa, respectively, according to the CBS strains database. Visagie et al. (2016) also observed inconsistencies between the individual phylogenetic trees forITS, BenA, CaM, and RPB2, respectively, for the *Torulomyces* sect. (*P. lagena* and *P. riverlandense*). Assuming that the unique placement of *P. alfredii*, *P. lagena*, and *P. riverlandense* in our analysis is true, it opens the possibility that they diverged from the *Penicillium* spp. prior to the divergence of the two subgenera *Penicillium* and *Aspergilloides* and thereby form the basis for a new subgenus. However, further studies regarding the matter should be investigated to support this suggestion.

With the increasing accessibility of sequencing data, pangenomic analyses are becoming more widespread in comparative genomics. A pangenome is a visualization of all available genomic information of a group of organisms to get a better understanding of the genomic makeup of a genus or species. Pangenome analyses are more common for bacteria and plants but recently some studies of pangenomes in fungi have been published as well (Barber et al. 2021; Wyka et al. 2022; Nielsen et al. 2017; Wang et al. 2021). To our knowledge, this is the first study that investigates a fungal pangenome of an entire genus with such a large number of high-quality de novo assemblies. By revealing the genomic diversity of a genus, variations and common characteristics of numerous species can be assessed in a relatively simple way. The pangenome can therefore function as an atlas to provide an easy overview of a genus.

Inherent to the lack of precise methods to identify true gene models, and relying on probabilistic predictions, artefact gene predictions are very likely to be observed and result in an inflated cloud genome. To assess the extent of this, we assessed the proportion of BUSCO genes observed in the cloud genome (20.6% completeness equaling to 742 genes) but 669 of these were fragmented, supporting that the major proportion of the cloud genome consists of gene fragments. In general, the choice of prediction algorithm and their tendency for overprediction may also contribute to artefactual inflation of the cloud genome. However, we observed that FunGAP (based on Braker, Maker, and Augustus (Min et al. 2017)) gene prediction on *P. digitatum* PDW03 leads to 9464 gene models, which is quite similar to the 9003 gene models of the original publication, using Braker alone (Wang et al. 2021).

Nielsen et al. (2017) presented a pangenome from 24 *Penicillium* species and found 3249 core genes and 8784 accessory genes. From their analysis, the pangenome appeared closed, meaning that including additional members to the group would provide few new additional genes and, hence, gene functions. However, members from the subgenus *Penicillium* were overrepresented (20 species) compared to subgenus *Aspergilloides* (four species). In contrast, we do not observe a closed pangenome, but indeed expected signs of saturation of the pangenome. In our dataset, the distribution of *Penicillium* (53 isolates) and *Aspergilloides* (51 isolates) are comparable, and species were selected to represent a wide diversity. We believe that the increased diversity causes the number of observed core and accessory genes to increase to 5612 and 24,607, respectively. However, we cannot exclude that our pangenome was based on a stricter alignment parameter (minimum percent match cutoff of 75% vs. 50%). The relatively open pangenome in this study indicates that, even though we have sampled a broader diversity of the genus compared to Nielsen et al. it is not sufficient to capture all the genetic variation of *Penicillium*. Of course, given the extremely high microbial diversity on Earth, with diversity estimates ranging from millions to trillions, it might not be practically feasible to sample and thereby capture all, or even nearly all, of the genetic information of a genus. However, the concept will provide an indication of the end of the scale. Thus, we find that conducting the analysis on groups of genes sorted according to size reveals that larger genes are approximating saturation faster than smaller genes. Assuming that shorter gene models are more likely to include non-functional pseudo-genes and gene fragments than rather genes, we hypothesize that the larger gene analysis is more likely to represent the true diversity. However, we cannot rule out that larger genes in general are under higher purifying selection than shorter, which may also impact this result. Further, comparison to the pangenome obtained by Nielsen et al. (2017) found that the KOG category responsible for secondary metabolism was mainly encoded in the core genome, whereas our analysis showed that it was predominantly in the accessory genome. This again is likely caused by some orthogroups involved in secondary metabolism being categorized as core genes due to the non-representative and limited diversity sample in the study by Nielsen et al. (2017). In this study, increasing the sampled diversity resulted in the placement of secondary metabolite genes as categories in the accessory genome, which is to be expected, considering the considerable literature available on diversity of observed secondary metabolites themselves across *Penicillium* (e.g., Houbraken et al. 2020).

Overall, the *Penicillium* pangenome reconstructed in this study seems reasonable with 5,612 core genes. Given the average gene count is 11,976 per genome, this infers that approximately half of all genes are shared between the *Penicillium* isolates. The result can form a solid basis for further comparisons between *Penicillium* isolates.

It is well known that LGT plays an important role in the evolution of fungi (Fitzpatrick 2012). For example, an early LGT analysis of *S. cerevisiae* S288C showed it has acquired 13 bacterial genes and these have contributed to important functional innovations, including the ability to synthesize biotin and the ability to grow under anaerobic conditions (Hall et al. 2005). One of the most infamous incidences of LGT relates to the acquisition of a toxin gene (ToxA) by *Pyrenophora tritici-repentis* from *Stagonospora nodorum* resulting in *Pyrenophora* infestations of wheat (Friesen et al. 2006). There are also multiple studies showing that several metabolic gene clusters, whose functions are often associated with fungal virulence, have experienced LGT (Campbell et al. 2012; Greene et al. 2014). In this study, we analyzed the genomes of 104 *Penicillium* genomes (71 unique species) to determine the frequency of recent bacterial lateral gene transfers into the *Penicillium* genus. Overall, we located 15 gene transfer events, encompassing 139 genes into the genus. Overall, this accounts for ~ 0.01% of the total protein dataset utilized here and is significantly lower than the 2.9% and 3.5% reported in other fungal species (Murphy et al. 2019; Wisecaver et al. 2014), but similar to comparisons previously undertaken in the CTG clade (Fitzpatrick et al. 2008). However, our analysis ignores more ancient bacterial to fungal transfers that may have occurred before the differentiation from other closely related genera in the Eurotiales order, or indeed even more ancient gene transfer events. Furthermore, we have not accounted for recent/ancient fungal-to-fungal gene transfer events, so the overall component of the dataset that has arisen through LGT will be larger. A previous LGT study utilizing 23 unique species of *Penicillium* located 60 LGT events into the genus (Wang and Ruan 2020). However, only seven of these (termed HGT1,3,18,26,45,53,56) LGTs were unique to the genus as the remainder were located in closely related fungi and most likely acquired before speciation between phyla and genera. Our analysis successfully recovered three of these gene *Penicillium* specific transfers (HGT1,18,56,\* MERGEFORMAT Table 1). We did not recover HGT1,3,18 because the proteins were either below our sequence length or similarity cutoff (see Methods). HGT26 was also discarded as there was an ortholog in *Aspergillus sydowii* (tree not shown) and it is therefore not deemed to be specific to the *Penicillium* genus. The frequency (32%) of genes with introns in the 139 putative LGT genes is lower than that observed in the *Penicillium* dataset (76.9%) and is consistent with their bacterial origin, as previously reported (Wang and Ruan 2020). Overall, the retention or loss of transferred genes varies. For example, the LGT2 event occurred at the last common ancestor to 15 species (21 genomes) and has been maintained by all of these (Figure S25). A similar pattern is observed for LGT7 where the gene transfer has occurred before the speciation of 28 unique species and has been maintained in all 36 representative genomes. Conversely, LGT1 and LGT8 are LGTs that occurred before the speciation of most of the *Penicillium* species considered here; however, their retention is very patchy and most likely associated with niche adaptation (Wang and Ruan 2020).

## CONCLUSION

In this study, we have sequenced, assembled, and annotated 93 *Penicillium* isolates. A combined *Penicillium* pangenome was generated with these genome assemblies together with eleven previously published *Penicillium* genome models. The pangenome consist of a core genome containing 5612 genes and a larger accessory genome of 24,607 genes, elucidating a diverse pangenome, likely with a high potential of producing a wide range of secondary metabolites. Analysis of the phylogenetic relationship based on shared BUSCO genes of the 104 *Penicillium* isolates largely confirmed previous results obtained with three maker genes. Finally, we identified 15 lateral gene transfer events from bacteria that have occurred during evolution of *Penicillium*.

## Supplementary Information

**Additional file 1**. Overview of isolates and software versions used in the assembly process.

**Additional file 2**. Additional figures S1-S25.

**Additional file 3**. GO enrichment.

**Additional file 4**. dN/dS.

**Additional file 5**. LGT.

**Additional file 6**. Orthogroups.

## Data Availability

Data for this study have been deposited at NCBI under the BioProject number PRJNA867151. Individual references for genome assemblies and their reads can be found in Additional file 1: Table S1. The *Penicillium* pangenome structed in orthogroups can be found in Additional file 6.
