# Supplementary material for: Comparative genomic study of the Penicillium genus elucidates a diverse pangenome and 15 lateral gene transfer events
Source: IMA Fungus. 2023 Feb 1;14:3. doi: 10.1186/s43008-023-00108-7 (PMC9893605; doi:10.1186/s43008-023-00108-7)
Supplement: Supplementary file 2 — Additional file 2. Additional figures S1-S25. [file 43008_2023_108_MOESM2_ESM.pdf]

Additional figures

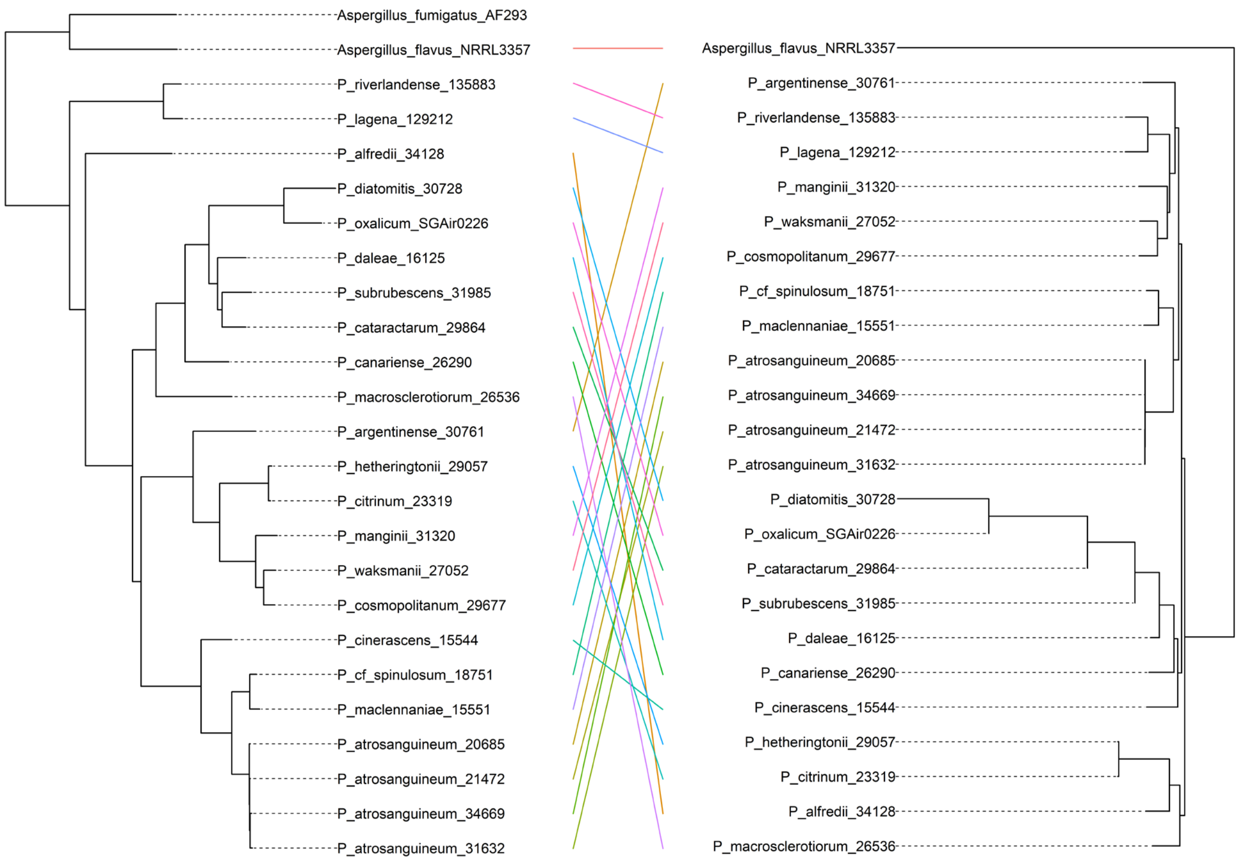

Figure S1: Comparison of phylogenies. The phylogeny based on conserved BUSCO gene families were pruned into four individual phylogenies. The tree on the left side is the first phylogeny part from the orginal phylogeny based on conserved BUSCO gene families. Each member in this tree were aligned again using information from the entire genome sequences in CLC workbench producing the tree on the right. Lines between the two trees indicates the placement of an isolate. The outgroup is only presented here due to clear division. Negative branches for *P. cataractrum*, *P. oxalicum*, and *P. subrubescens* in the right tree were charged to zero.

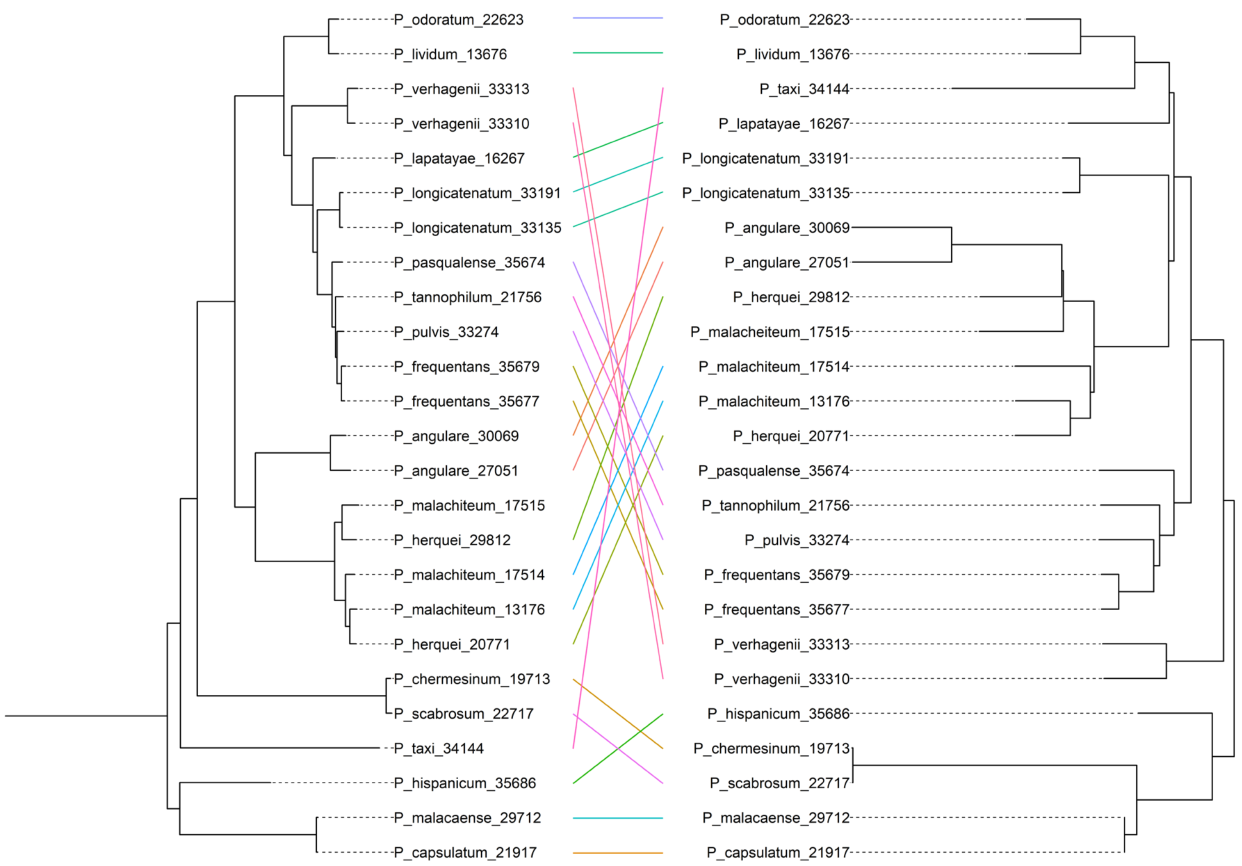

Figure S2: Comparison of phylogenies. The phylogeny based on conserved BUSCO gene families were pruned into four individual phylogenies. The tree on the left side is the second phylogeny part from the orginal phylogeny based on conserved BUSCO gene families. Each member in this tree were aligned again using information from the entire genome sequences in CLC workbench producing the tree on the right. Lines between the two trees indicates the placement of an isolate.

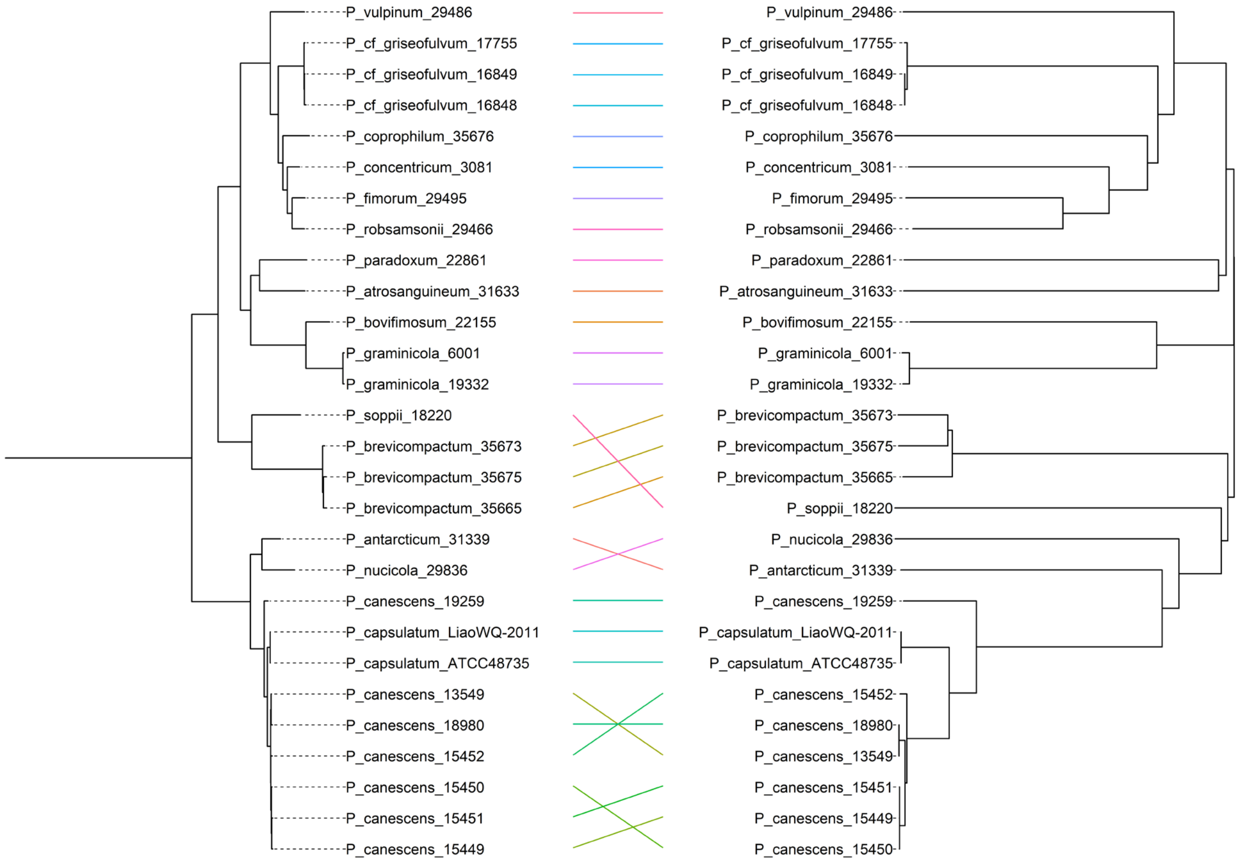

Figure S3: Comparison of phylogenies. The phylogeny based on conserved BUSCO gene families were pruned into four individual phylogenies. The tree on the left side is the third phylogeny part from the orginal phylogeny based on conserved BUSCO gene families. Each member in this tree were aligned again using information from the entire genome sequences in CLC workbench producing the tree on the right. Lines between the two trees indicates the placement of an isolate.

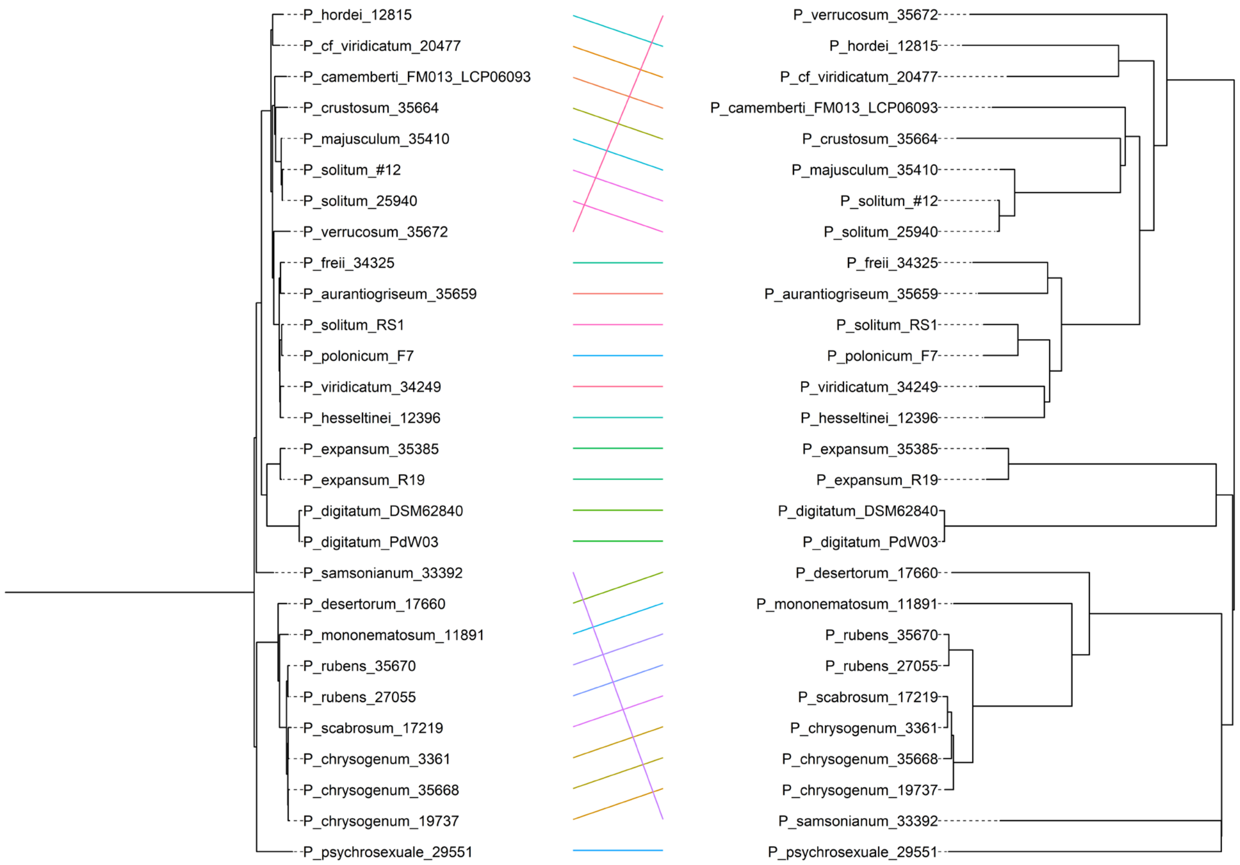

Figure S4: Comparison of phylogenies. The phylogeny based on conserved BUSCO gene families were pruned into four individual phylogenies. The tree on the left side is the fourth phylogeny from the orginal phylogeny based on conserved BUSCO gene families. Each member in this tree were aligned again using information from the entire genome sequences in CLC workbench producing the tree on the right. Lines between the two trees indicates the placement of an isolate.

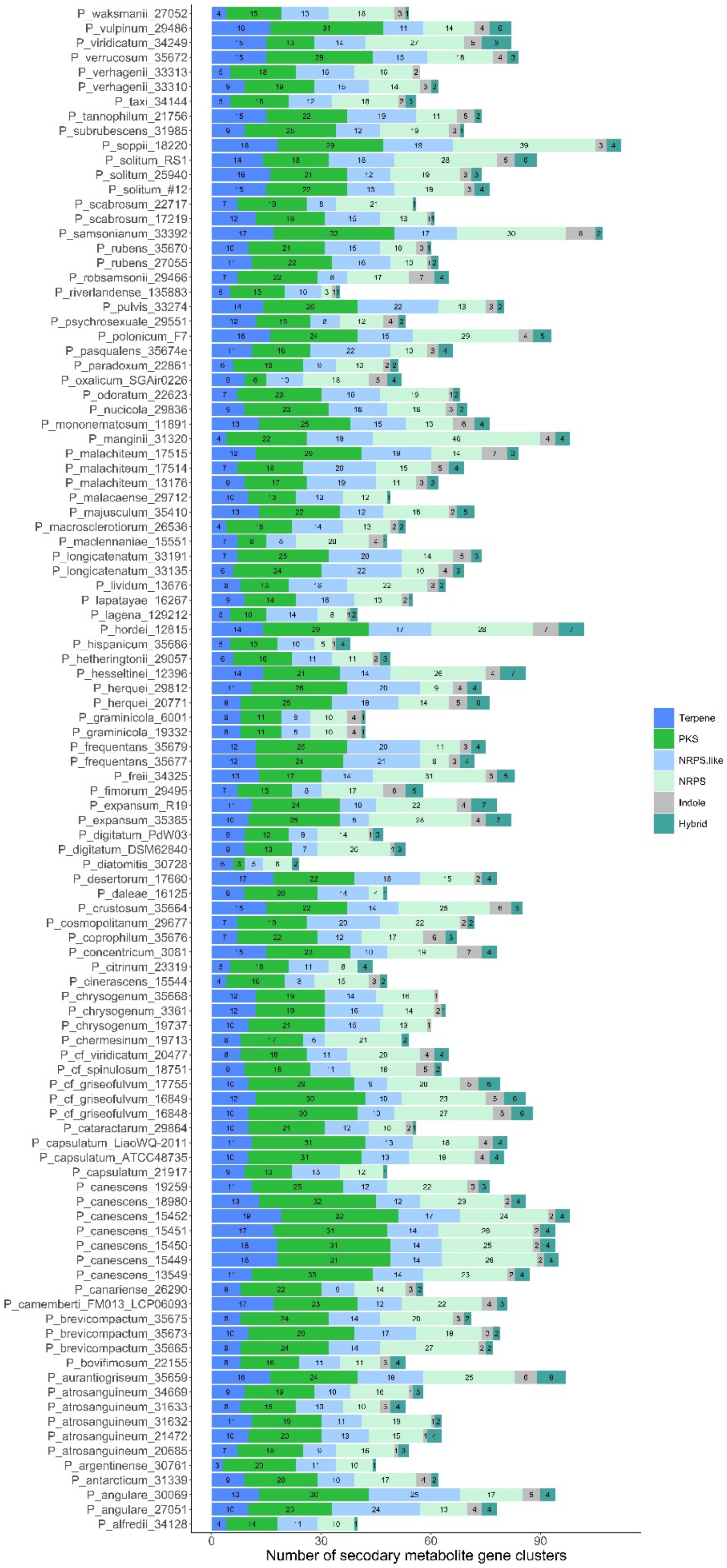

Figure S5: Secondary metabolites profiles of core genes in biosynthesis gene clusters. Gene prediction obtained from FunGAP was used as input to antiSMASH for each genome draft.

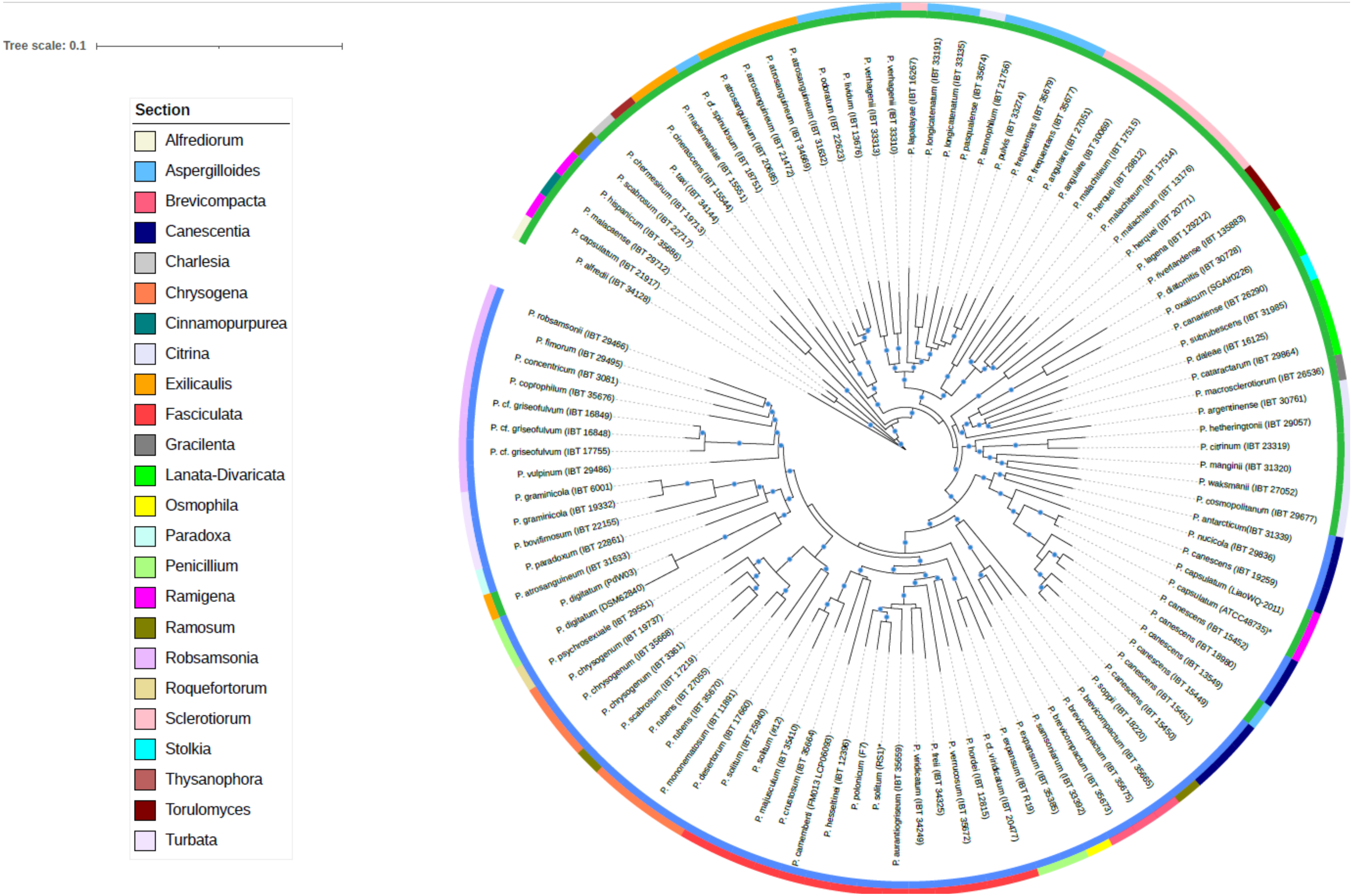

Figure S6: Representation of the phylogenetic relationship between the 104 Penicillium isolates. The phylogeny is based on present or absent of all orthogroups in the pangenome. The inner circle is colored according to the subgenera whereas the outer circle is colored according to sections as described in Houbraken et al. [1]. The blue circles states nodes with a bootstrap value of ≥95%. The tree scale is amino acid substitutions per site.

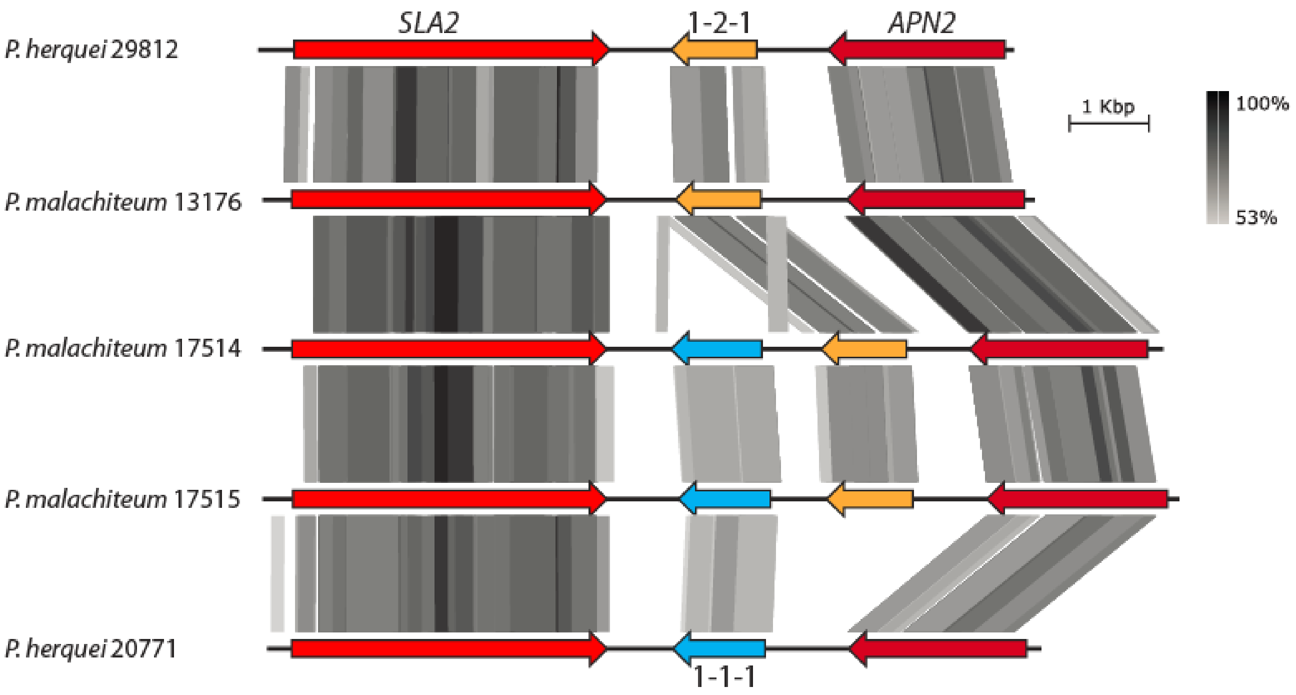

Figure S7: Synteny plot comparing five mating loci reveal two out of three *P. malachiteum* strains carry both *MAT1-1-1* and *MAT1-2-1* mating type genes. The genes illustrated with red, yellow, blue, and dark red encode the SLA2 gene, the MAT1-2-1 gene, the MAT1-1-1 gene and the APN2 gene, respectively. The grey bars show tBLASTx identity score between nucleotide sequences; darker tone signifies higher identity percentage. Mapping reads back to the assemblies of *P. malachiteum* (IBT 17514) and *P. malachiteum* (IBT 17515) showed single reads spanning the entire mating loci. This validates the observed structure of the mating loci in the assemblies.

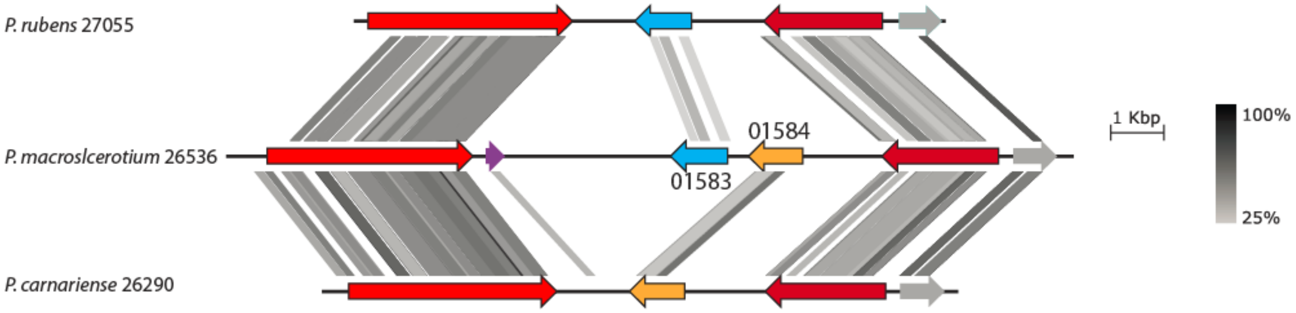

Figure S8: Synteny plot revealing *P. macroscleroteum* (IBT 26536) carries both *MAT1-1-1* and *MAT1-2-1* mating type genes. The genes illustrated with red, yellow, blue, and dark red encode the SLA2 gene, the MAT1-2-1 gene, the MAT1-1-1 gene and the APN2 gene, respectively. The grey bars show tBLASTx identity score between nucleotide sequences; darker tone signifies higher identity percentage. The locus comprises an additional gene, Pmacroscleroteum_01582 showed in purple, which shares homology to the gene Pdiatomitis_09181 and outside any ORF in *P. carnariense* (IBT 26290). Mapping reads back to the assemblies of *P. macroscleroteum* (IBT 26536)) showed single reads spanning the entire mating loci. This validates the observed structure of the mating loci in the assemblies.

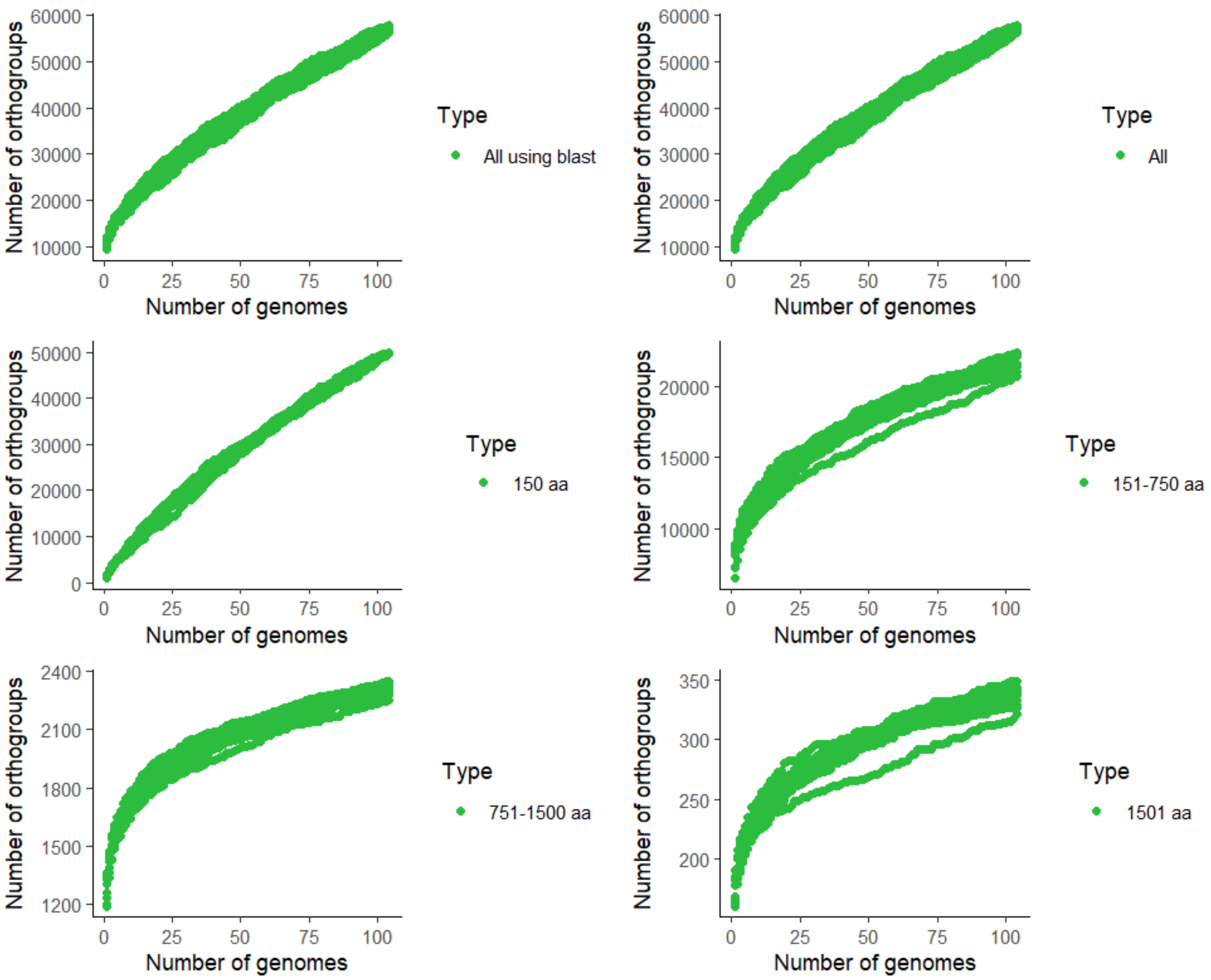

Figure S9: The cumulative size of the Penicillium gene pool in subset. The subsets were divided according to protein sequence length. Blast was used in the similarity search for the data “all using blast” whereas Diamond was used in the similarity search in the rest.

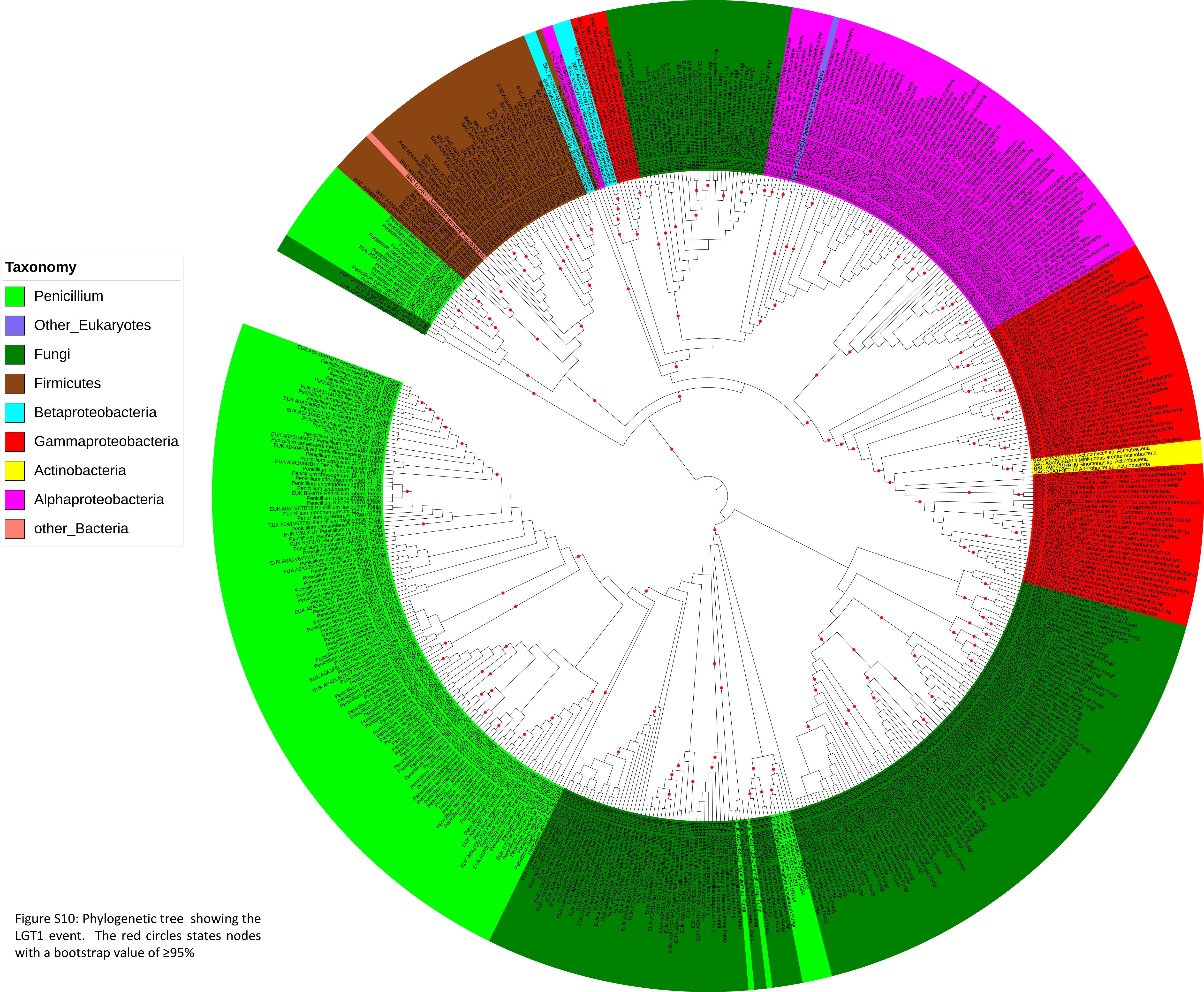

Figure S10: Phylogenetic tree showing the LGT1 event. The red circles states nodes with a bootstrap value of ≥95%

Figure S11: Phylogenetic tree showing the LGT2 event. The red circles states nodes with a bootstrap value of ≥95%.

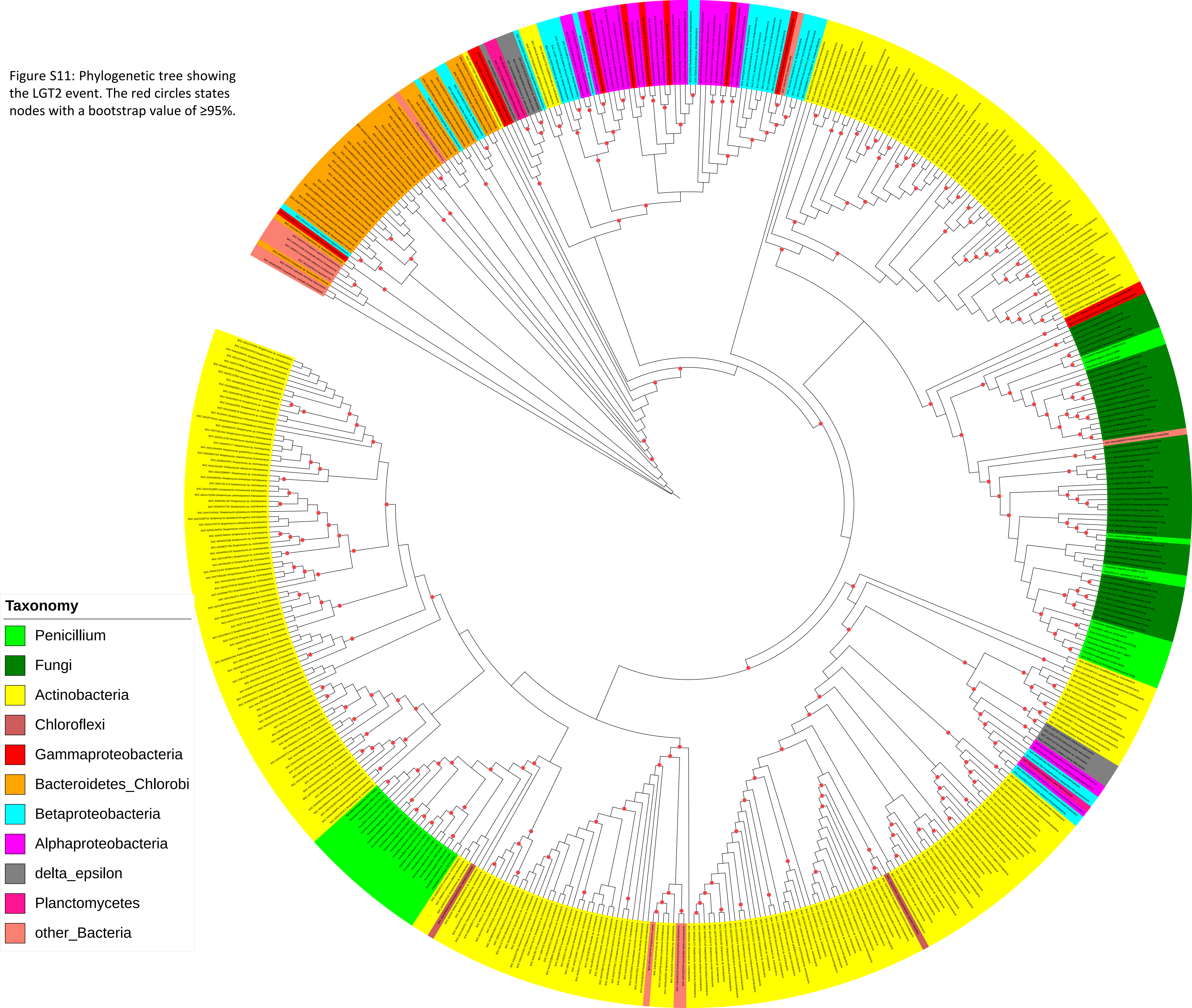

Figure S12: Phylogenetic tree showing the LGT3 event. The red circles states nodes with a bootstrap value of ≥95%.

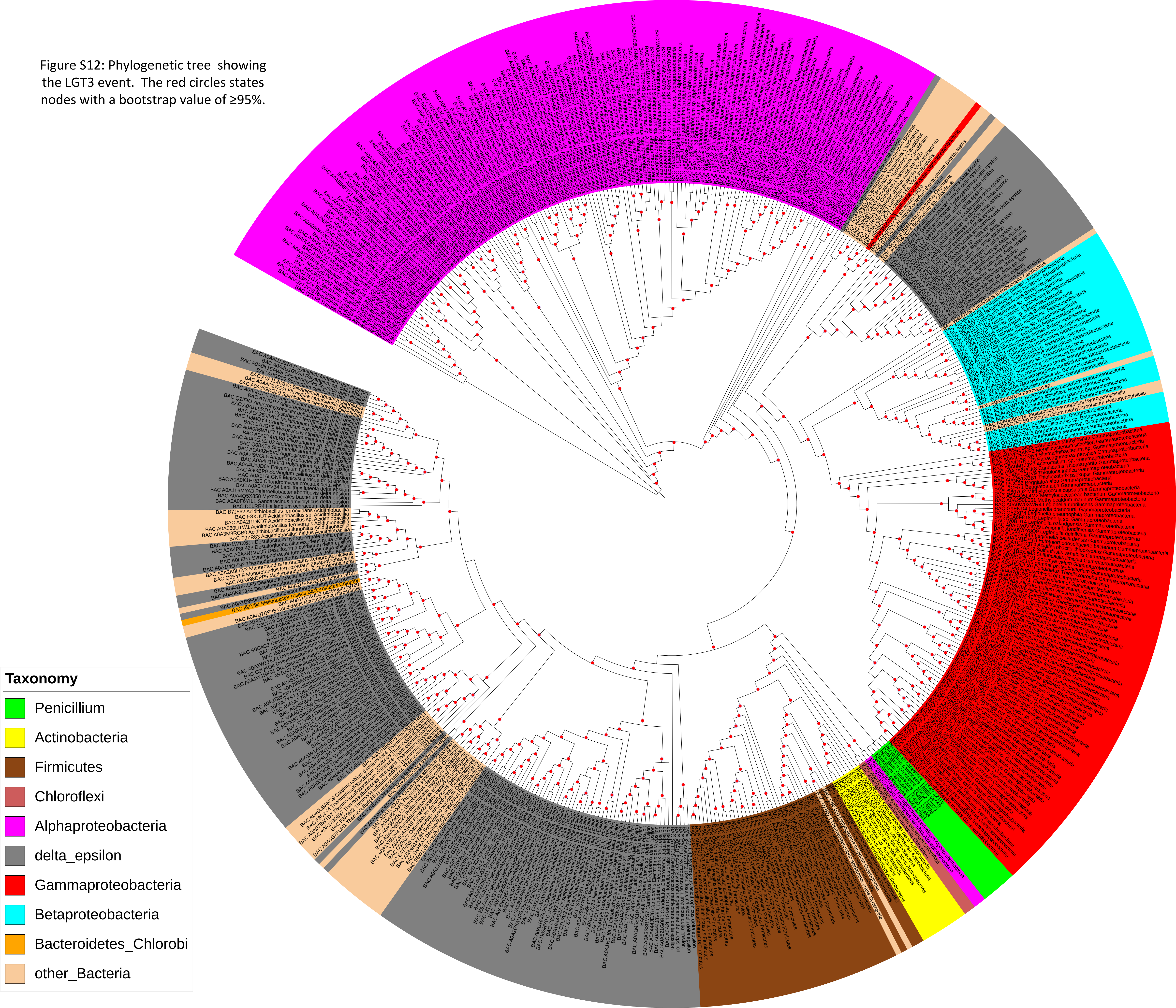

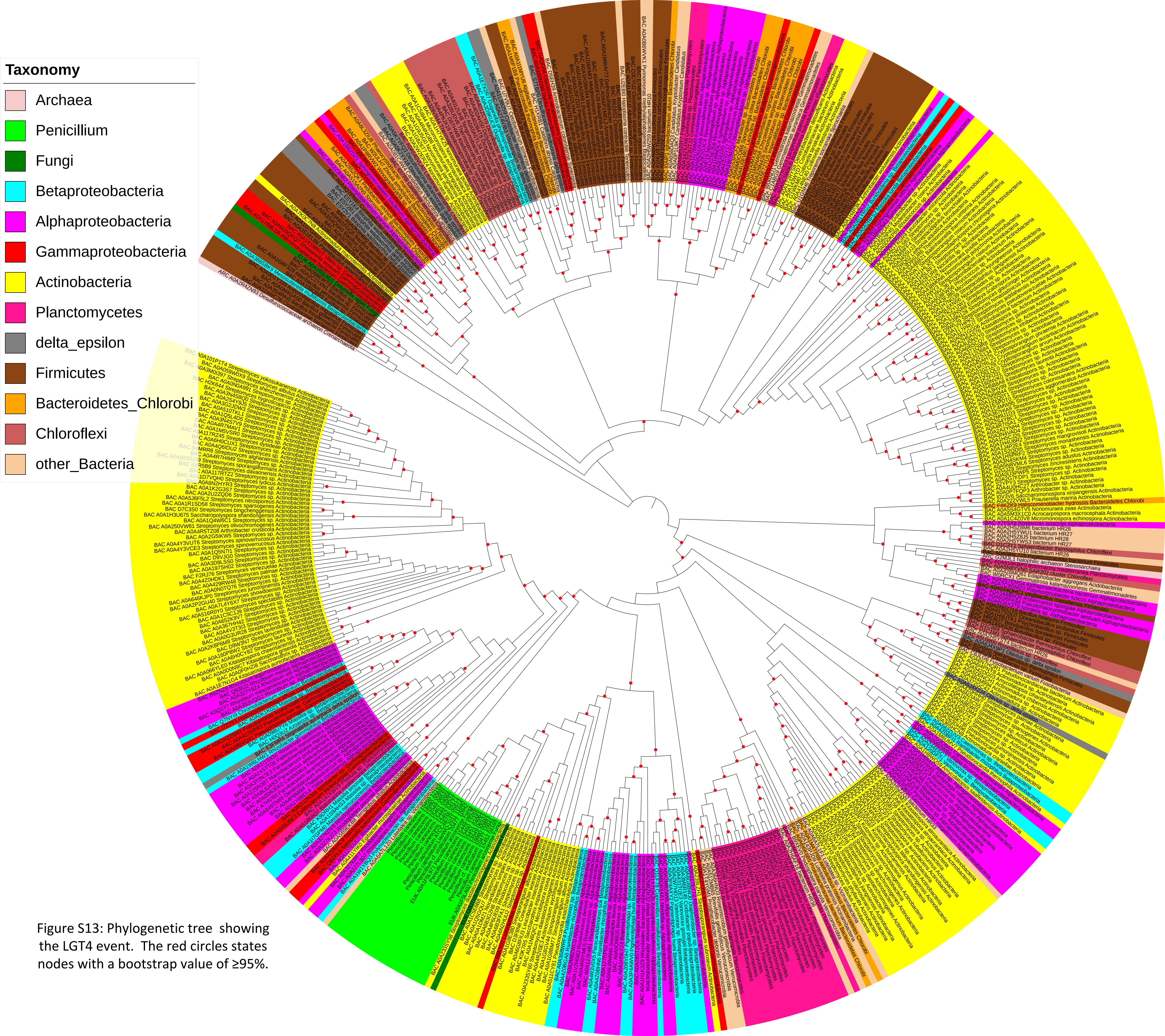

Figure S13: Phylogenetic tree showing the LGT4 event. The red circles states nodes with a bootstrap value of ≥95%.

Figure S14: Phylogenetic tree showing the LGT5 event. The red circles states nodes with a bootstrap value of ≥95%.

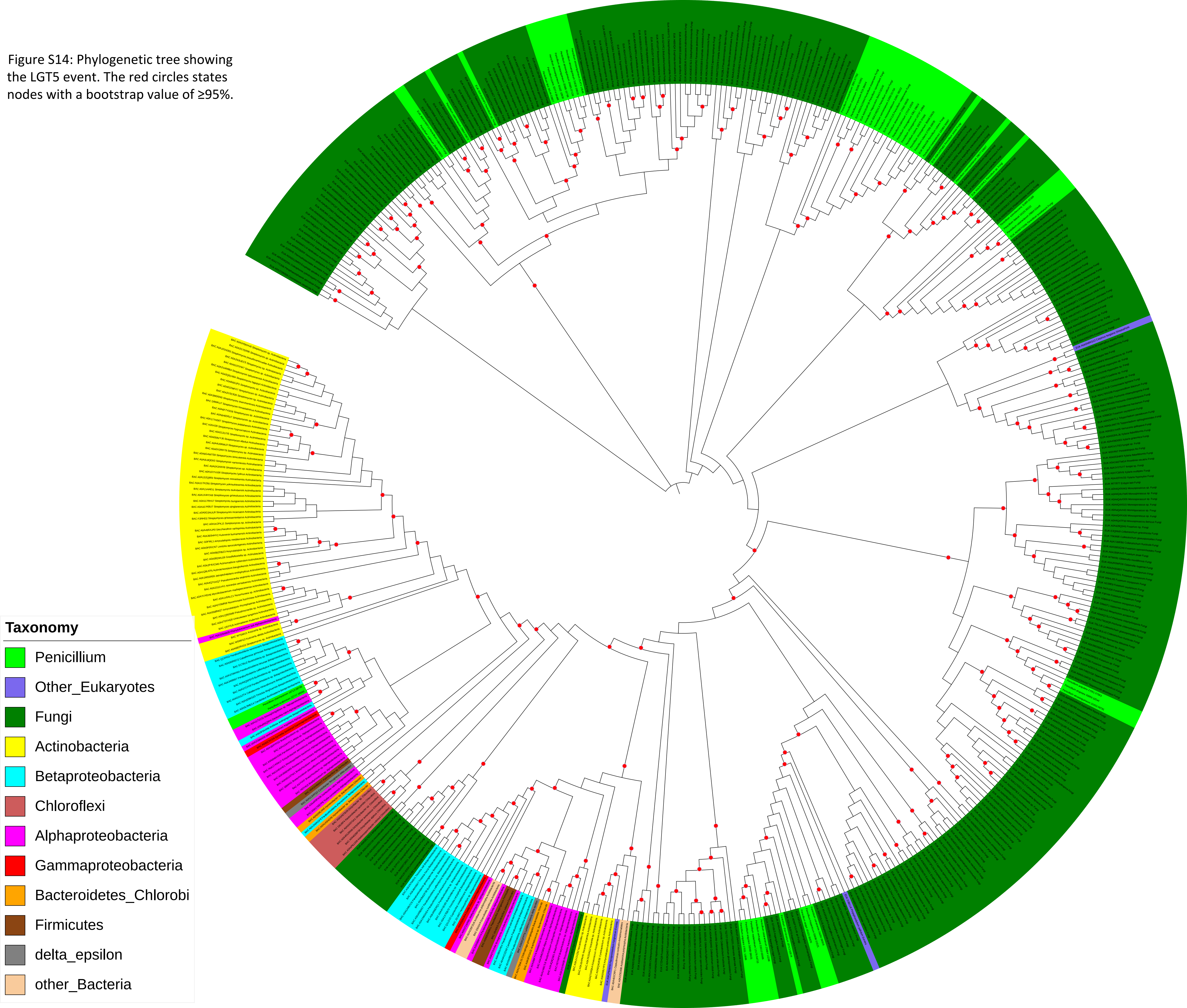

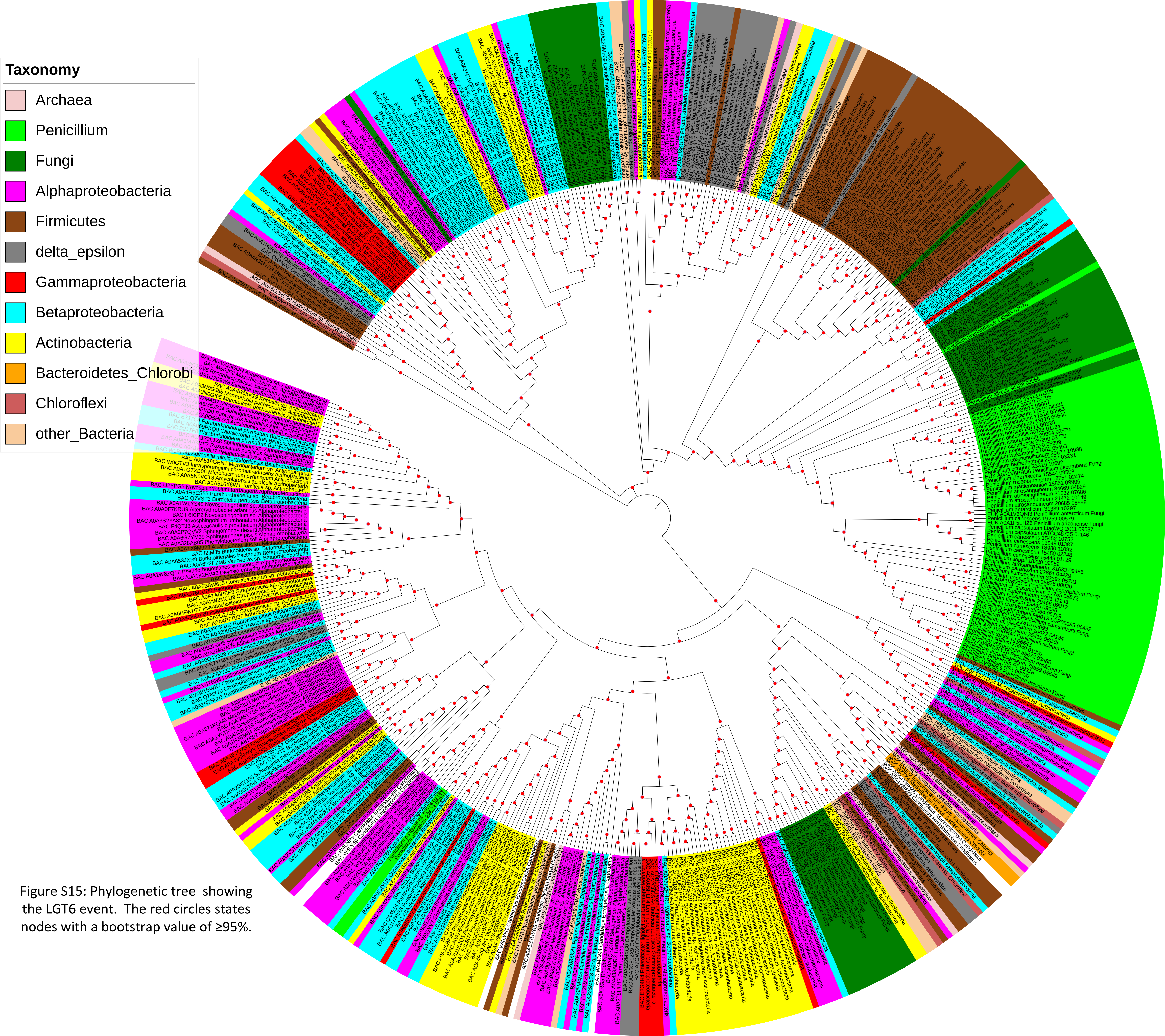

Figure S15: Phylogenetic tree showing the LGT6 event. The red circles states nodes with a bootstrap value of ≥95%.

Figure S16: Phylogenetic tree showing the LGT7 event. The red circles states nodes with a bootstrap value of ≥95%.

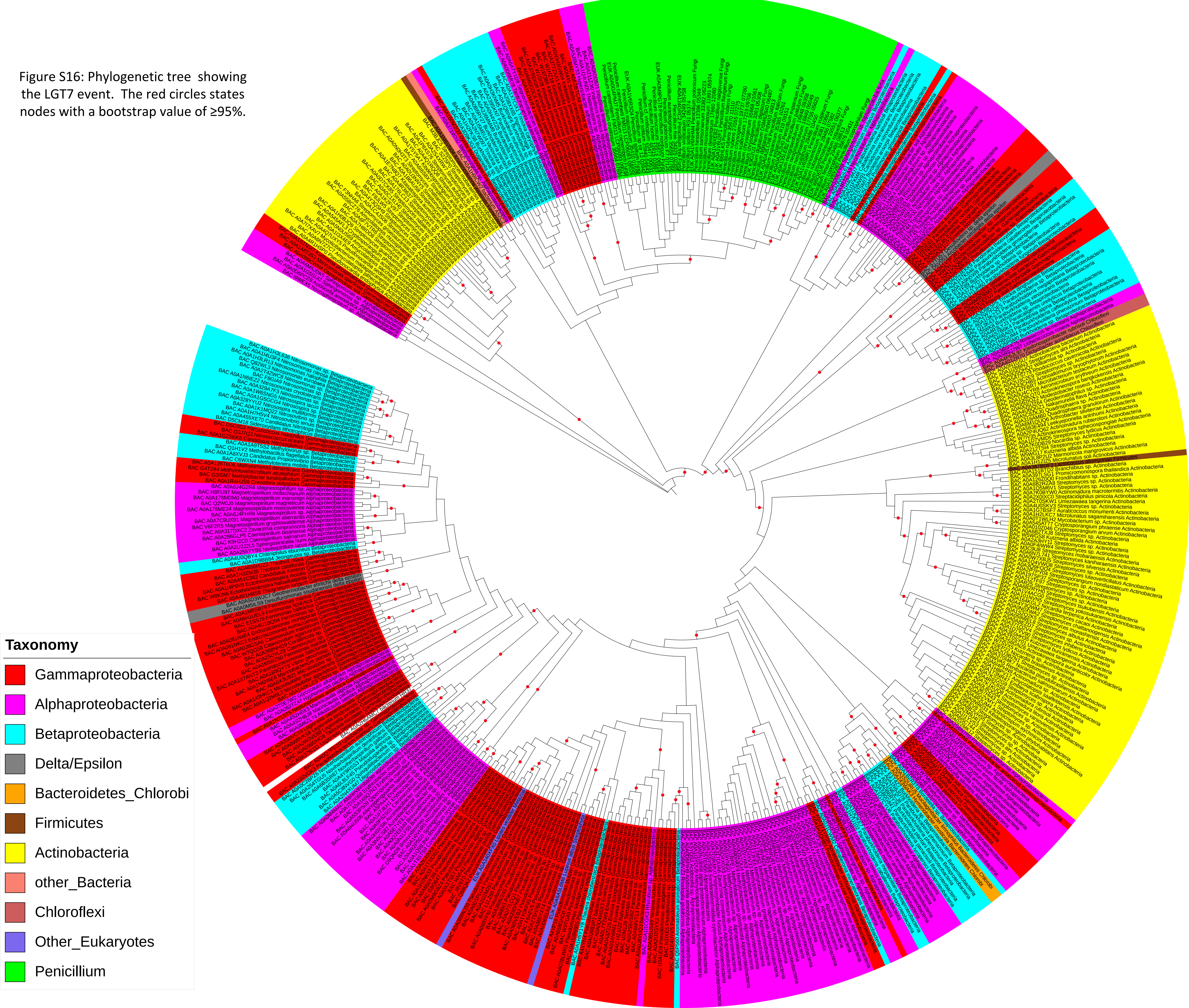

Figure S17: Phylogenetic tree showing the LGT8 event. The red circles states nodes with a bootstrap value of ≥95%.

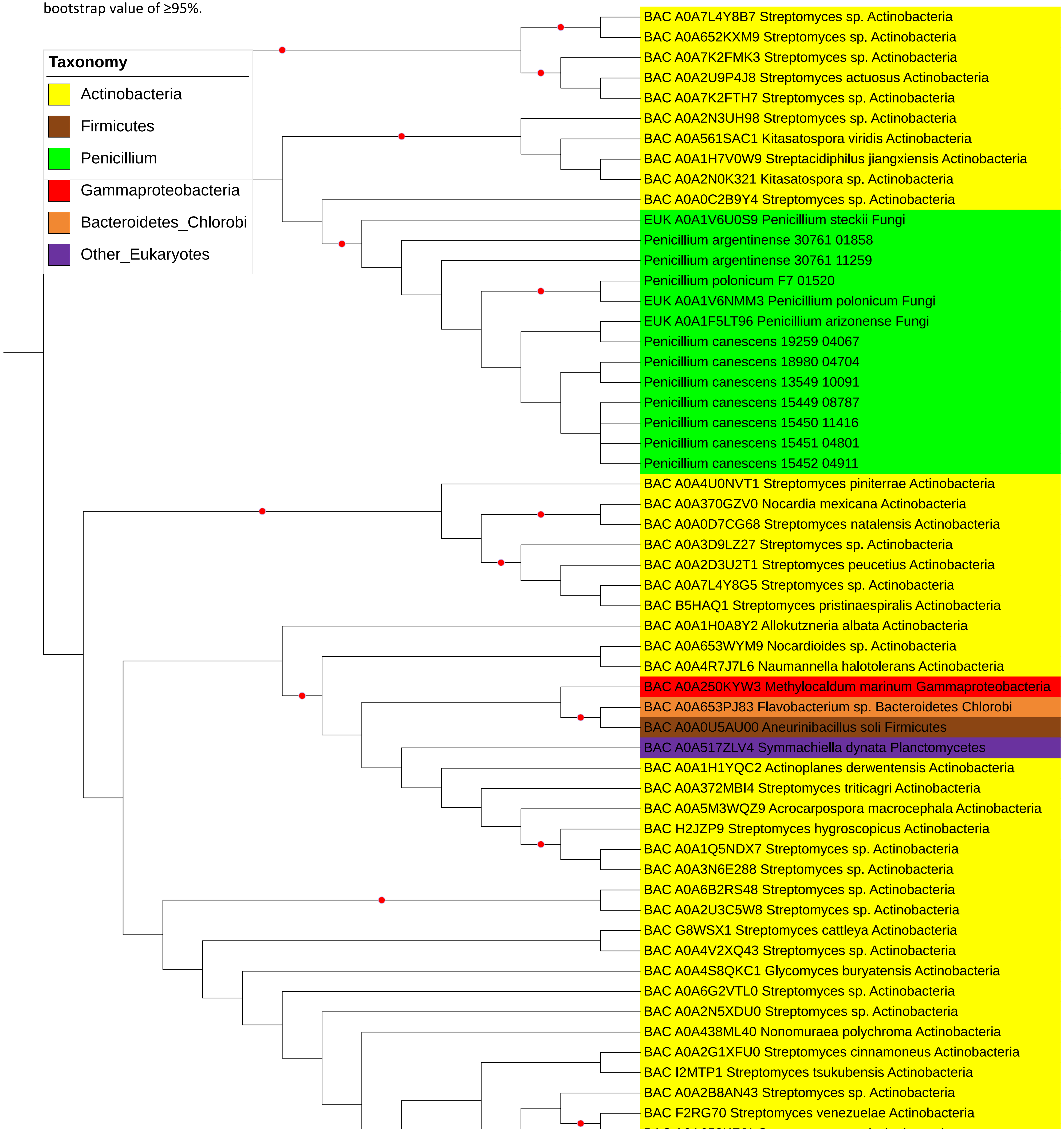

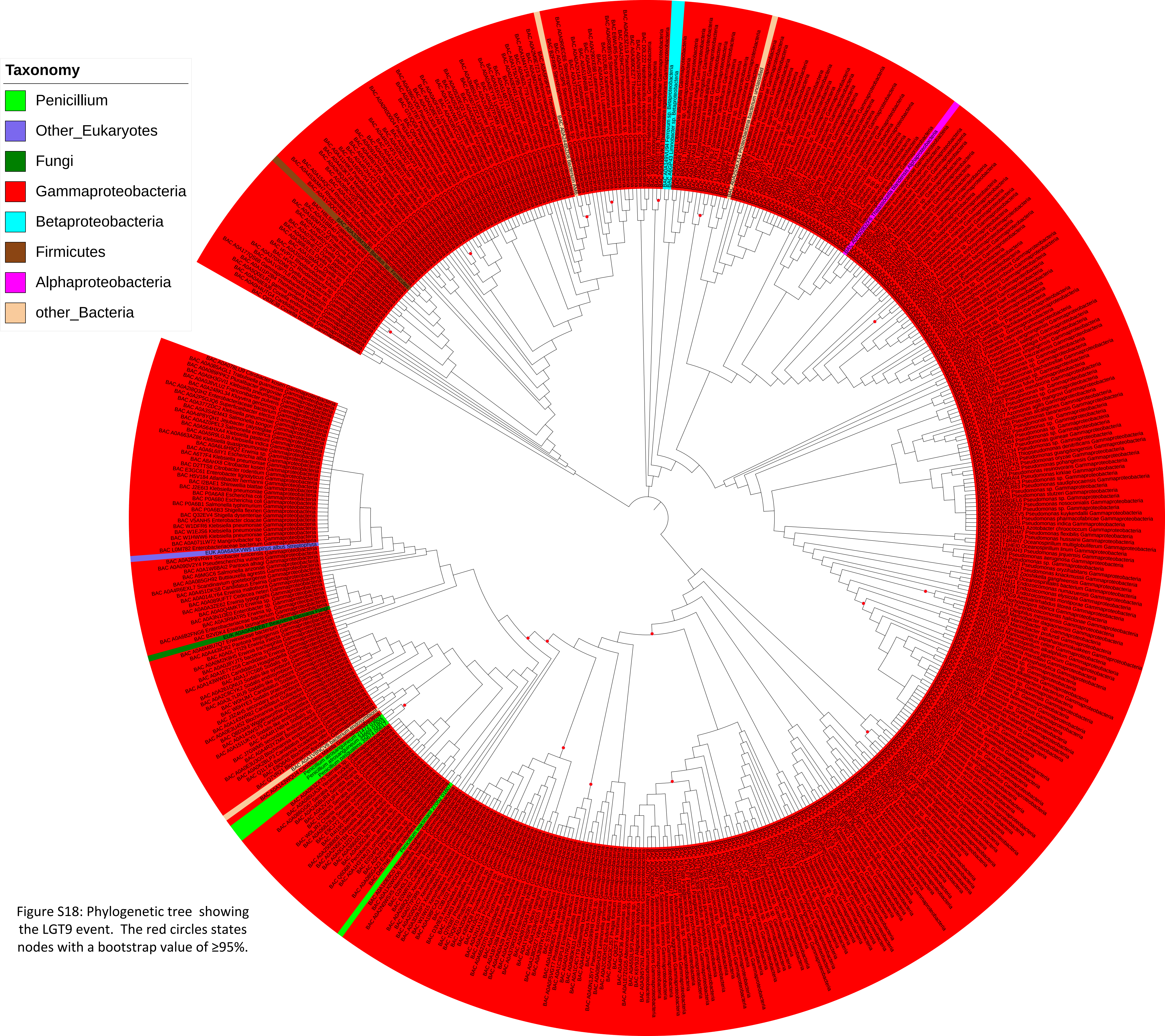

Figure S18: Phylogenetic tree showing the LGT9 event. The red circles states nodes with a bootstrap value of ≥95%.

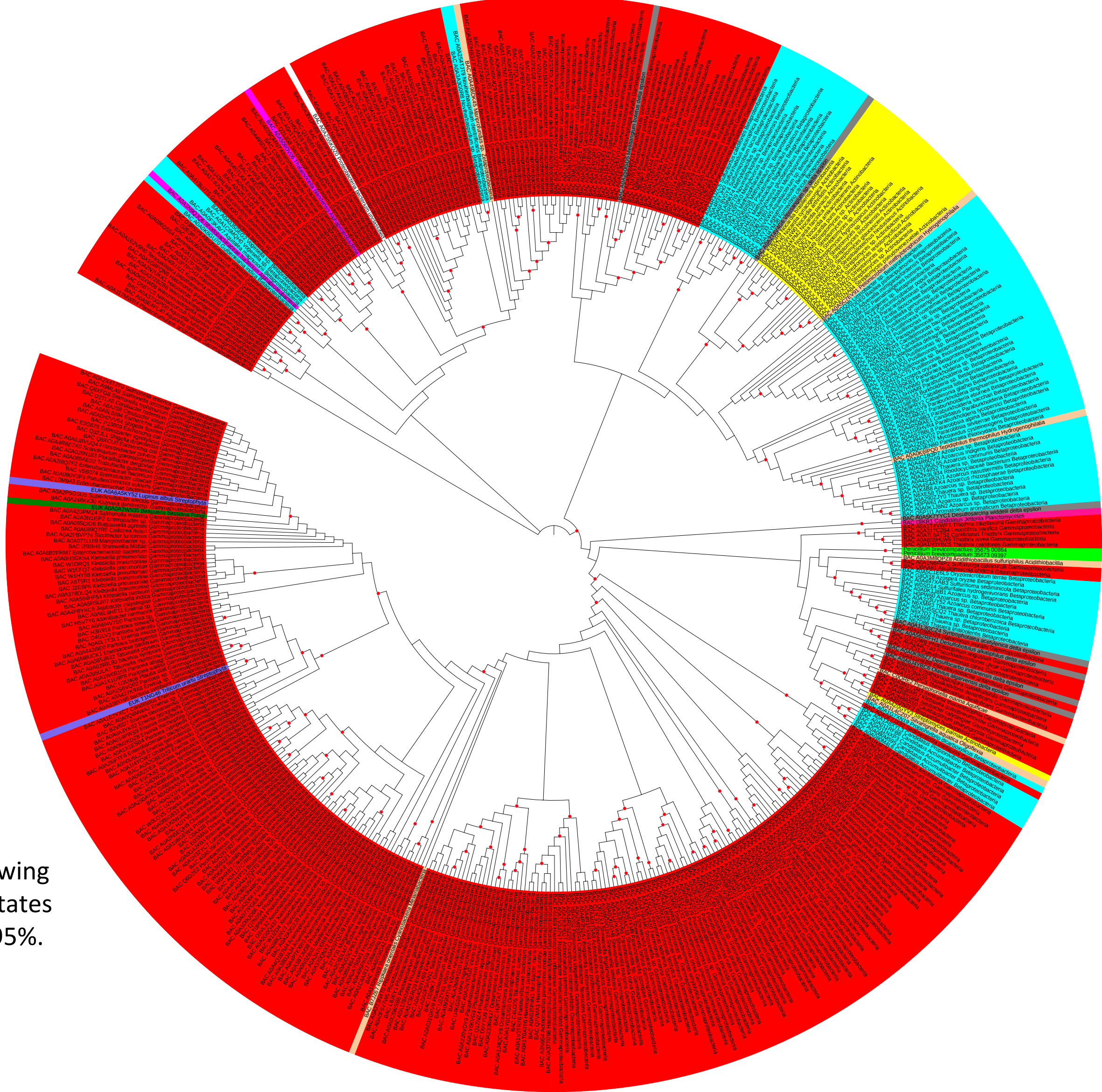

Figure S19: Phylogenetic tree showing the LGT10 event. The red circles states nodes with a bootstrap value of ≥95%.

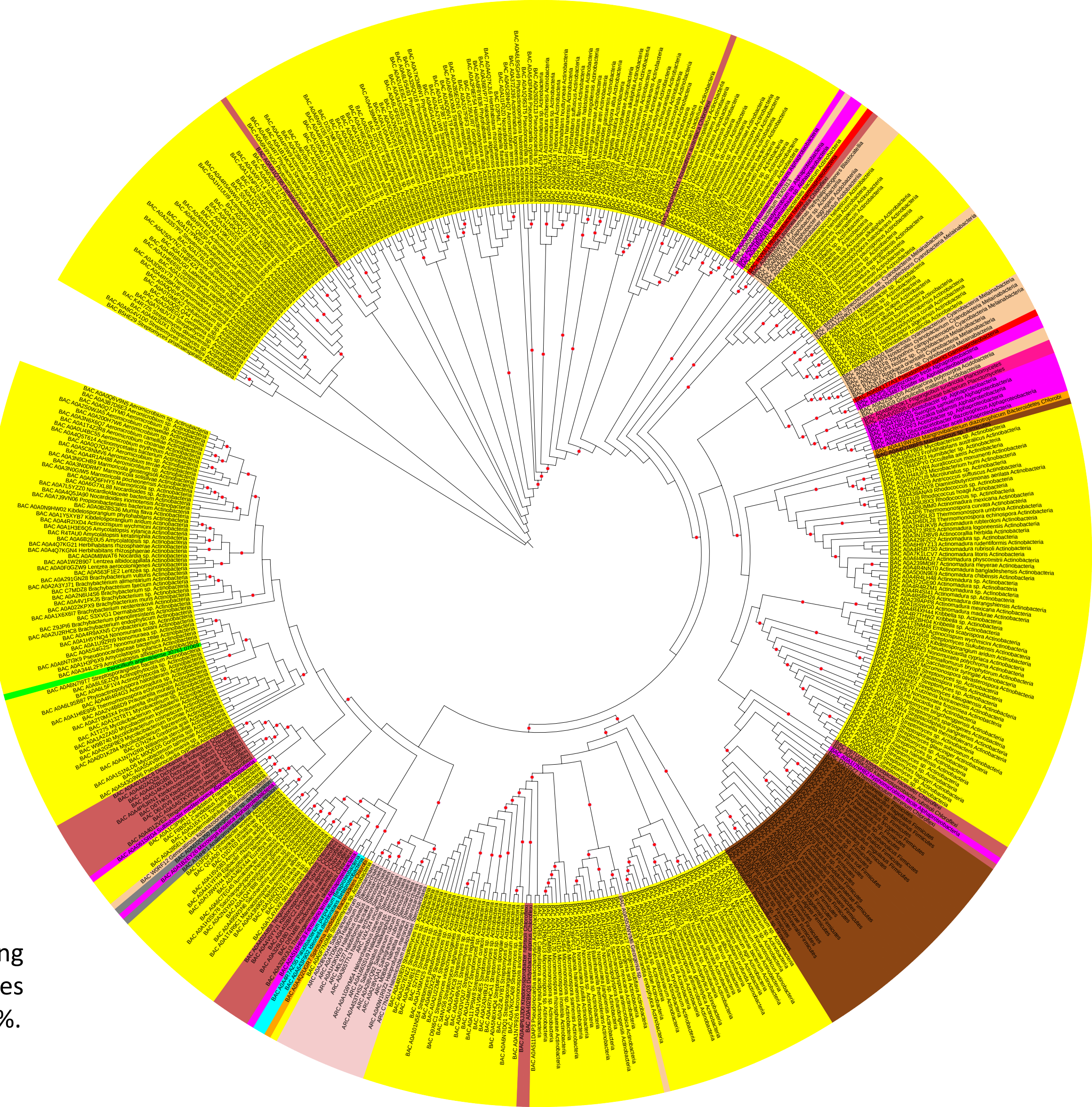

Figure S20: Phylogenetic tree showing the LGT11 event. The red circles states nodes with a bootstrap value of ≥95%.

Figure S21: Phylogenetic tree showing the LGT12 event. The red circles states nodes with a bootstrap value of ≥95%.

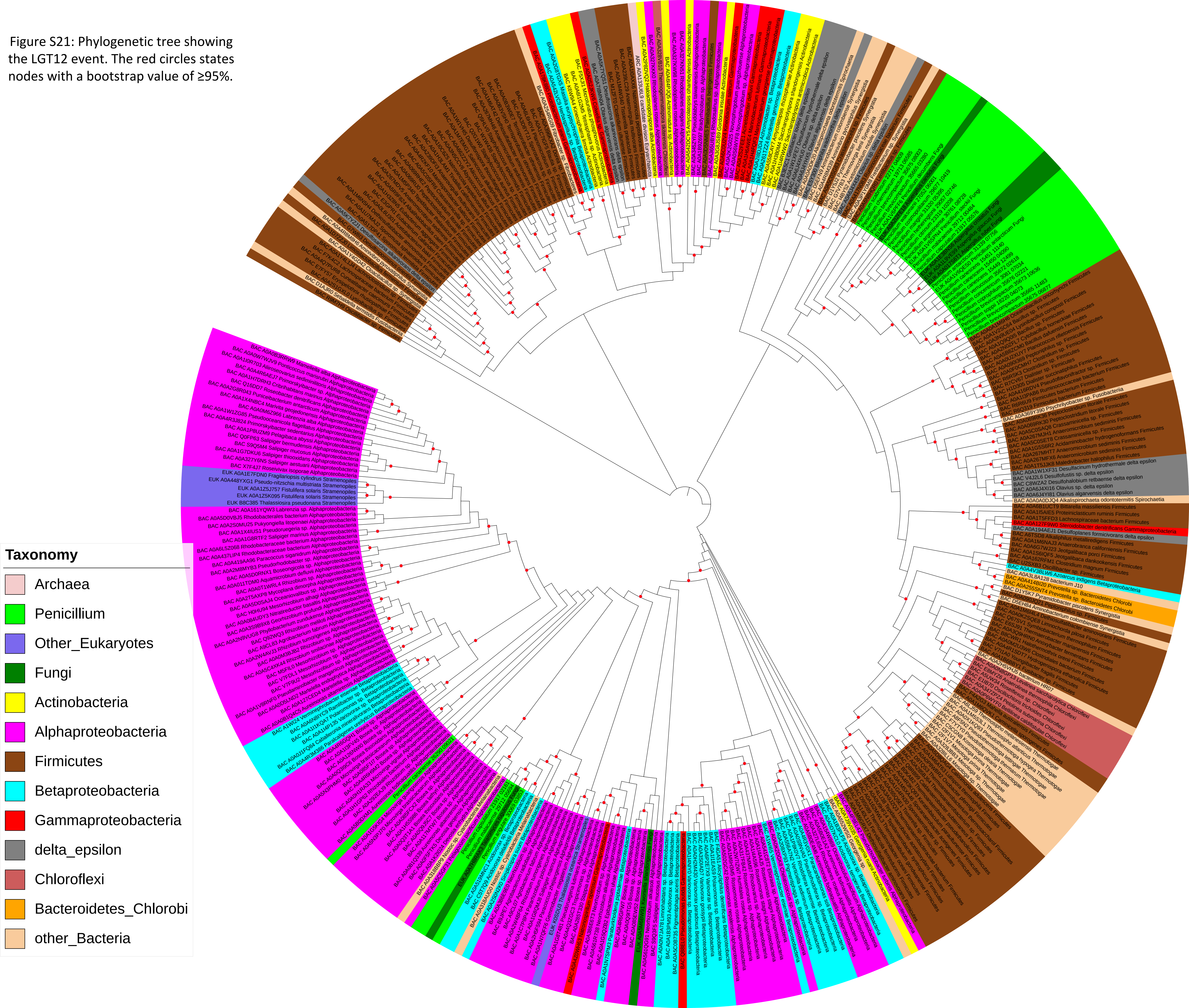

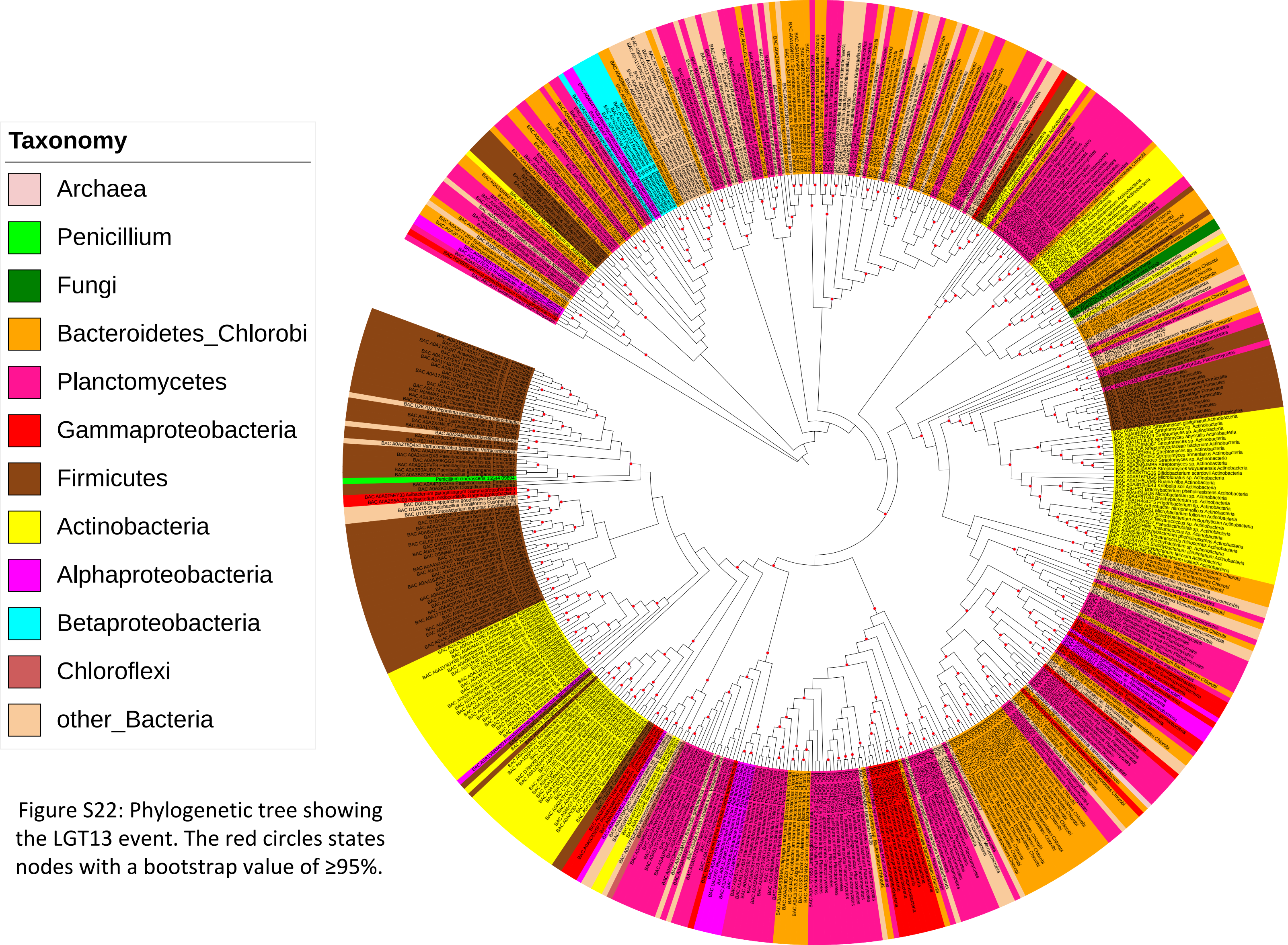

Figure S22: Phylogenetic tree showing the LGT13 event. The red circles states nodes with a bootstrap value of ≥95%.

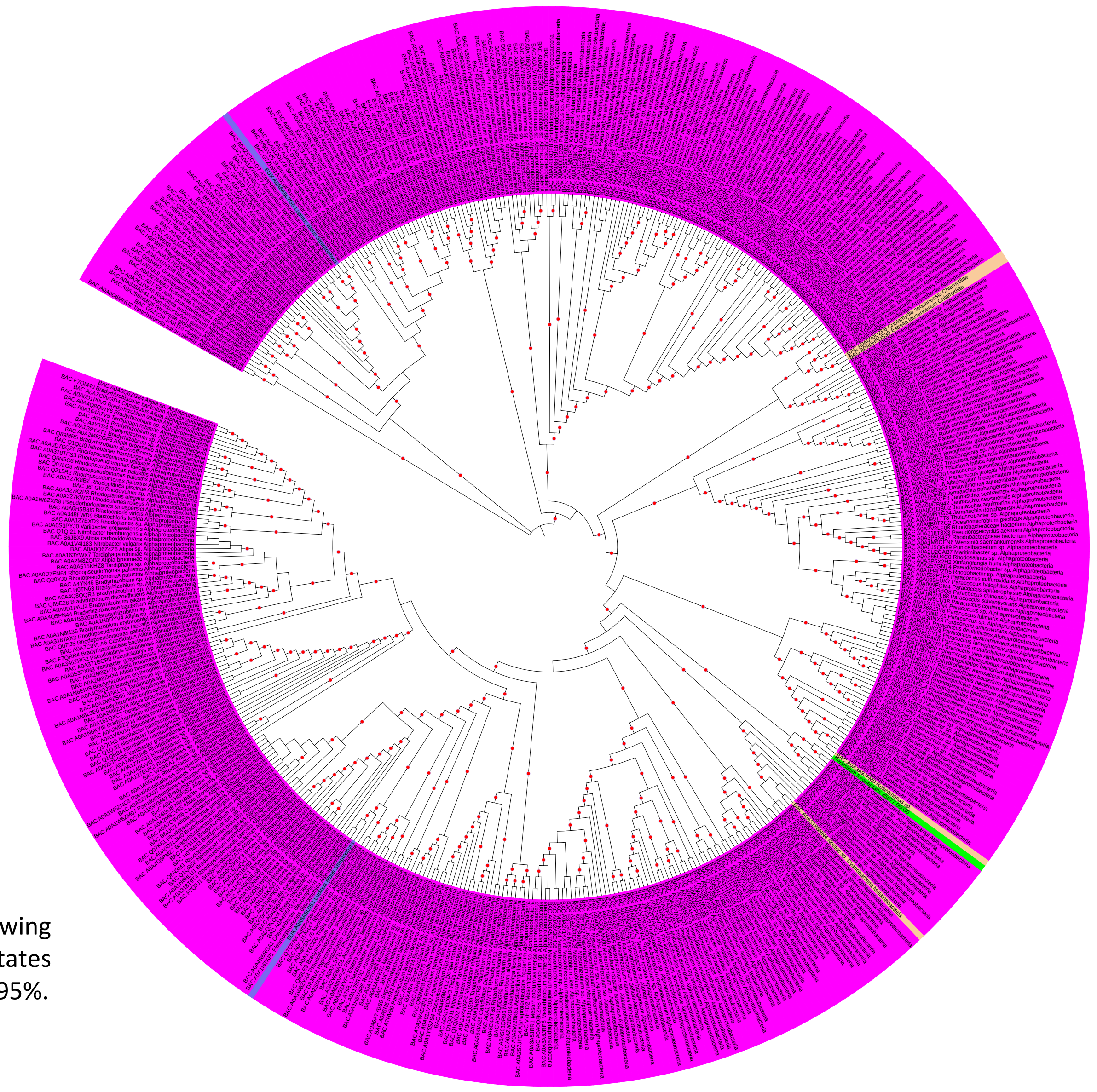

Figure S23: Phylogenetic tree showing the LGT14 event. The red circles states nodes with a bootstrap value of ≥95%.

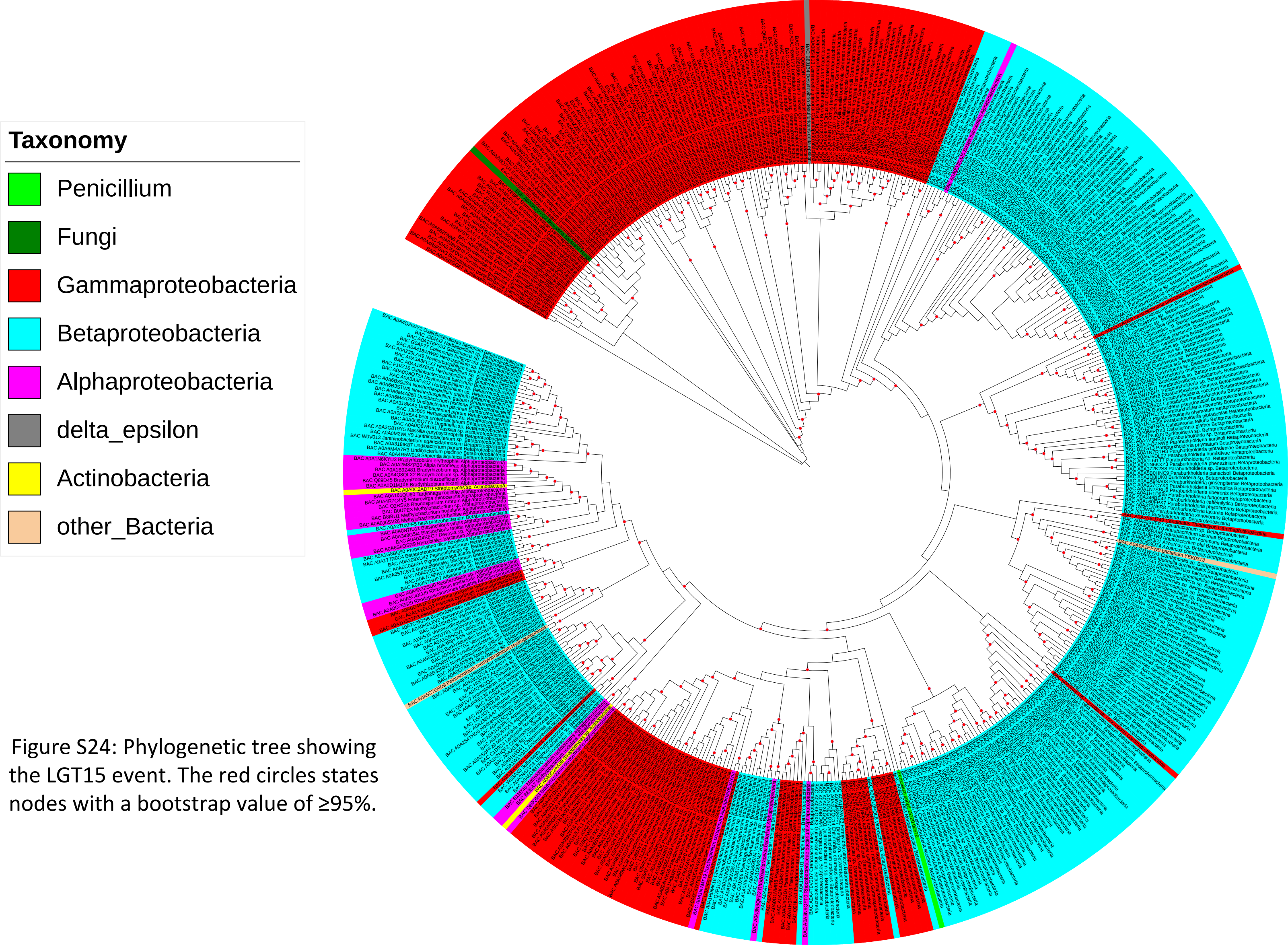

Figure S24: Phylogenetic tree showing the LGT15 event. The red circles states nodes with a bootstrap value of ≥95%.

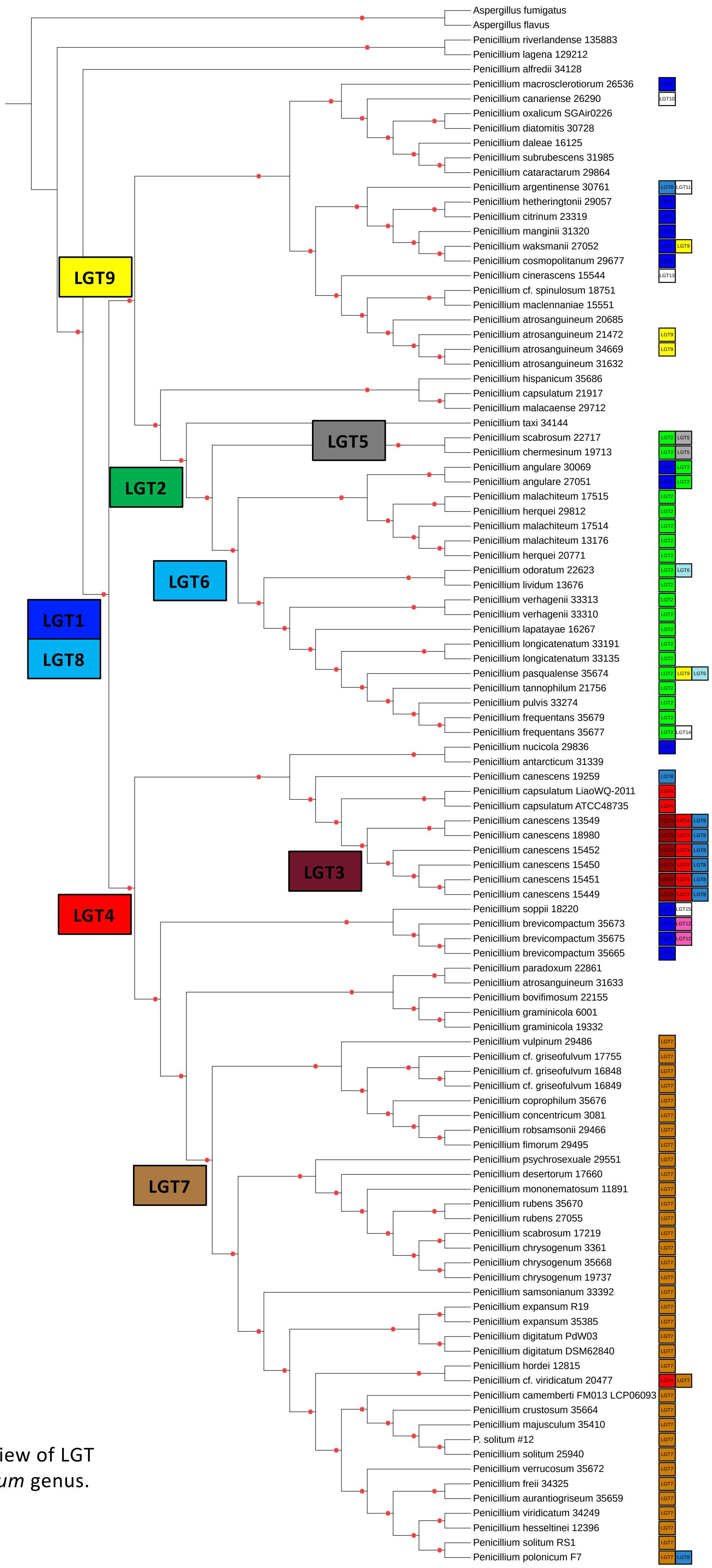

Figure S25: Overview of LGT events in *Penicillium* genus.
